# Knowledge, attitudes, and practice in breast cancer early detection: evidence from a nationally stratified study of healthcare providers in Jordan

**DOI:** 10.3389/fpubh.2026.1845992

**Published:** 2026-07-22

**Authors:** Husam ALSalamat, Ehsan Qtaishat, Dina Jardaneh, Mai Al-Hadid, Reem Al-Ajlouni

**Affiliations:** 1 Jordan Breast Cancer Program, King Hussein Cancer Foundation, Amman, Jordan; 2 Independent Researcher, Amman, Jordan

**Keywords:** breast cancer screening, early detection, healthcare providers, Jordan, KAP

## Abstract

**Background:**

Breast cancer remains the most prevalent cancer among women in Jordan. Although national early detection efforts have expanded over recent years, opportunities remain to further improve timely diagnosis and screening uptake. Healthcare providers (HCPs) play a central role in influencing screening uptake, and strengthening their knowledge, attitudes, and practices (KAP) can support program effectiveness.

**Objective:**

To assess KAP of HCPs related to breast cancer early detection in Jordan and identify factors influencing their translation into practice.

**Methods:**

A nationally stratified cross-sectional study was conducted among 544 HCPs between January and June 2025 using a structured, interviewer-administered questionnaire. Composite scores for KAP were calculated and further dichotomized for multivariable logistic regression to identify predictors of KAP domains.

**Results:**

The mean knowledge score was 57.0%, with high knowledge of signs and symptoms (78.1%) but substantially lower knowledge of screening recommendations, including clinical breast examination (27.4%) and mammography guidelines (28.6%). Attitudes were generally favorable (53.5%), with strong professional orientation (82.2%) and high perceived patient-level barriers (77.3%). Mean practice score was reflecting moderate implementation (49.0%). While most providers reported encouraging screening (98.9%) and appropriate case management (91.9%), correct screening practices were limited, including initiation age (30.3%) and screening intervals (20.8%). Female providers demonstrated higher odds of adequate knowledge (OR 1.78) and optimal practice (OR 1.93), while nurses had significantly lower knowledge (OR 0.46) and practice (OR 0.17). Regional disparities were observed, with lower knowledge but higher practice in southern regions.

**Conclusion:**

The findings indicate opportunities to better align provider knowledge, attitudes, and routine practice, particularly in screening-related competencies. Strengthening role-specific training, integrating guidelines into routine care, and addressing implementation and service-delivery factors are essential to improve early detection outcomes in Jordan and similar settings.

## Introduction

Breast cancer remains one of the leading causes of morbidity and mortality among women worldwide and continues to pose a significant public health challenge in Jordan. According to the 2023 National Cancer Registry, it is the most common cancer among women in the country, accounting for 38.3% of all female cancer cases and 20.7% of total cancer cases across all genders (1).

Detecting breast cancer at earlier stages remains one of the most effective ways to improve survival and reduce the overall burden of disease. In Jordan, the Jordan Breast Cancer Program JBCP supported a range of national efforts to support early detection, including awareness campaigns, community outreach, provider training, and the expansion of screening services (2). While progress has been made, a proportion of cases continue to be diagnosed at later stages. At the same time, emerging evidence suggests that breast cancer in Jordan is being identified at younger ages and at later stages compared with global trends, adding further complexity to both clinical management and public health planning (3). Together, these patterns suggest that areas for further improvement may remain within early detection pathways, including coordination, access, and implementation processes.

Within this context, healthcare providers HCPs play a central role. Through their daily interactions with patients, they influence how women understand breast cancer, perceive their own risk, and decide whether to seek screening (4, 5). They also guide patients through referral pathways and help translate national recommendations into practice. Their knowledge of screening guidelines, attitudes toward early detection, and routine clinical behaviors therefore have a direct impact on how effectively early detection strategies are implemented. As such, understanding HCPs' readiness to support early detection is critical for strengthening breast cancer control efforts.

However, while considerable attention has been given to raising public awareness and improving access to services, there remains value in further understanding how HCPs are supported and equipped to deliver these services in routine practice. Evidence from different settings suggests that providers are often more confident in recognizing clinical signs and symptoms than in applying screening guidelines or programmatic recommendations (6–8). In addition, broader implementation challenges, such as limited integration of performance indicators into daily practice and variability in adherence to screening protocols, have been reported across health systems (9, 10). These findings highlight the need to better understand not only what providers know, but also how that knowledge is applied in practice and shaped by the service environments in which they work.

Existing Jordanian evidence is limited in scope, Alkhasawneh et al. (11) examined nurses, and Ayoub et al. (12) focused on community pharmacists. Neither examined a multi-professional nationally stratified sample, nor linked provider Knowledge, Attitudes, and Practices KAP to system-level performance indicators or guideline alignment. The present study addresses both gaps, a stratified national sample across the largest clinically active provider groups, and an explicit framing of provider knowledge against the national early-detection performance framework.

Accordingly, this KAP study was conducted among HCPs from multiple specialties to assess their understanding of breast cancer early detection, their attitudes toward its importance, and their routine clinical practices related to screening. The study also sought to identify gaps in screening-related knowledge, explore how knowledge and attitudes translate into practice, and examine factors associated with variations across KAP domains. In addition, it explored perceived patient- and system-level barriers that may be associated with the implementation of early detection activities. Beyond the national context, the study aims to provide insights that are relevant to similar healthcare systems in the region, where structured screening programs exist alongside ongoing implementation challenges. By bringing together provider-level and system-level perspectives, this work contributes to a more practical understanding of how to bridge the gap between awareness and effective implementation of early detection strategies in low- and middle-income settings.

## Methodology

### Study design and setting

This was a national stratified cross-sectional study conducted among HCPs in Jordan between January and June 2025 to assess their KAP regarding breast cancer early detection and screening. The study also explored perceived barriers and enablers influencing the integration of breast cancer screening and health promotion into routine clinical practice. Data were collected across multiple healthcare settings, including public hospitals, primary healthcare centers, comprehensive health centers, private hospitals, and private clinics, to capture variability in service delivery contexts. A structured, interviewer-administered questionnaire was used to obtain quantitative data on HCP engagement in breast cancer early detection.

### Study population and sampling

A multi-stage stratified design was used. Stratification was applied by region (North, Central, South) and professional category, with target allocations proportional to the distribution of HCPs in the Jordan Ministry of Health 2024 annual report (general practitioners 44.0%, nurses 52.0%, obstetrics and gynecology specialist 2.8%, family medicine 1.0%, community medicine 0.2%; total reference population 57,648) (13). Because some specialties had very small population sizes, sample boosting was applied for family medicine, gynecology, and community medicine to ensure analytical viability for sub-group analyses. Of a planned 525 participants, 544 were ultimately enrolled. Community medicine specialists could not be recruited within the data-collection window owing to the very small national workforce (planned *n* = 50; achieved *n* = 0). University hospitals and the Jordanian Royal Medical Services were not included because the required institutional approvals were not obtained within the field-work period. Recruitment in the private sector of the southern governorates was constrained by accessibility, partially explaining the lower achieved sample in that stratum. Recruitment was conducted in person at participants' workplaces by trained enumerators. Eligible HCPs encountered during enumerator visits were briefed on the study aims, procedures, voluntary nature of participation, and right to withdraw, and provided written informed consent prior to interview. No briefed eligible participant declined to consent during data collection; cooperation among briefed eligibles was therefore essentially complete. Approach-stage non-response, providers who were unavailable, on duty, or otherwise not encountered during enumerator visits, was not systematically enumerated by the field team. Sampling weights and finite-population correction were not applied; estimates reflect the achieved sample rather than the underlying provider population. Comparison of the achieved sample with the MOH reference distribution is provided in Supplementary Table S1.

### Study instrument

The instrument was developed in English by the Jordan Breast Cancer Program based on an extensive review of the literature and existing tools assessing HCPs' KAP related to breast cancer early detection and screening. The instrument underwent content validation through systematic review by subject matter experts in oncology, primary care, and public health to ensure relevance, clarity, and contextual appropriateness for the Jordanian healthcare setting.

The questionnaire included sections relevant to the outcomes presented in this study, covering: sociodemographic and professional characteristics; knowledge of breast cancer (including general knowledge, risk factors, and signs and symptoms); attitudes toward breast cancer screening and early detection, including perceived barriers; and practice related to the integration of breast cancer screening and health promotion into routine clinical care. The knowledge domain comprised 67 items assessing actual knowledge, in addition to 9 items assessing perceived knowledge (presented in Supplementary Table S2). The attitude domain included 28 Likert-scale items. Practice was assessed using 14 items, including 7 items related to clinical decision-making and 7 items assessing the frequency of clinical practices.

### Pilot testing and pretesting

Following expert review, the approved instrument was translated into Arabic and refined for clarity and cultural appropriateness, and the questionnaire was pilot tested among a small group of HCPs prior to field deployment to assess item comprehension, flow, and the functionality of skip logic within the digital tool; no major modifications were required, and pilot data were excluded from the final analysis. The instrument is therefore best described as content-validated and pilot-tested. Internal consistency was assessed *post hoc* and described later.

### Ethical considerations

The study was conducted in accordance with the ethical principles outlined in the Declaration of Helsinki and applicable national regulations. Ethical approval was obtained from the Institutional Review Board of the Jordan Ministry of Health (IRB Code: MOH REC/2024/102).

All participants were informed about the objectives of the study, the voluntary nature of participation, and their right to withdraw at any time without consequences. Informed consent was obtained from all participants prior to data collection. No personal identifiers were collected, and all data were anonymized to ensure confidentiality. Data were securely stored and accessed only by the research team.

### Data collection procedures

Data were collected through face-to-face interviews conducted with HCPs at their workplaces using trained enumerators. Each interview lasted approximately 30–35 min. Data collection was performed using a digital platform (KoboToolbox) on tablet devices, applying a computer-assisted personal interviewing approach.

The electronic questionnaire incorporated built-in validation rules, skip logic, and mandatory fields to enhance data completeness and accuracy during collection. Data were collected across healthcare facilities in different regions of Jordan to ensure coverage of the target population.

All required permissions were obtained through official communication with the Ministry of Health and relevant facility administrations prior to data collection. The study protocol was also ethically approved by the Ministry of Health in Jordan.

### Data quality assurance and cleaning

A structured quality assurance process was implemented throughout data collection and management. Enumerators received standardized training on the study objectives, questionnaire content, ethical considerations, and digital data collection procedures, including supervised practice interviews. Field supervisors monitored data collection, conducted random spot checks, and performed routine reviews of submitted questionnaires.

Data were reviewed in real time by the central research team to identify inconsistencies or incomplete entries, allowing timely feedback to field teams. Following completion of data collection, the dataset was cleaned by checking for completeness, consistency, and logical accuracy. The cleaned dataset was then imported into SPSS (version 31), with appropriate coding and labeling of variables prior to analysis.

### Variable definitions and scoring

Knowledge, attitude, and practice were assessed using composite measures derived from the questionnaire domains.

Actual knowledge was evaluated using 67 items covering general knowledge, risk factors, and signs and symptoms of breast cancer. Each item was scored based on predefined correct responses, and a total knowledge score was calculated by summing correct answers, with higher scores indicating better knowledge. Equal weighting of items across domains was applied as standard practice for KAP composite scoring in the absence of validated domain-specific weights, consistent with the approach used in comparable instruments in this field (7, 12). Correct responses for screening-related items (mammography starting age, stopping age, interval; CBE frequency; BSE frequency and starting age; role of ultrasound; expected national early- and late-stage detection rates) were defined in accordance with the national breast cancer screening and diagnosis guidelines, which is adapted with permission from the National Comprehensive Cancer Network NCCN 1.2017 Breast Cancer Screening and Diagnosis Guidelines (14). Specifically, mammography screening was scored as recommended from age 40 with no upper limit, at 1–2 year intervals; CBE annually; BSE monthly from age 25. Perceived knowledge was assessed separately using 9 items and presented descriptively.

Attitudes were assessed using 28 Likert-scale items, grouped into five domains: professional orientation toward breast cancer prevention (6 items), perceived public awareness (2 items), perceived patient-level barriers (9 items), system and structural barriers (8 items), and provider readiness and self-efficacy (3 items). Responses were recorded on a five-point scale ranging from strongly disagree to strongly agree and were numerically coded from 1 to 5. Items reflecting perceived patient-level and system-level barriers were reverse-coded for the calculation of the overall attitude score to ensure that higher scores consistently indicated more favorable attitudes toward breast cancer screening and early detection. Domain-specific subscale scores were calculated using original item coding to preserve construct-specific interpretation.

Practice was assessed using 14 items, comprising two domains: proper practice decisions (7 items) and proper practice frequency (7 items). For descriptive analysis, optimal and non-optimal practices were reported based on item-level classifications. Decision-based items were classified according to predefined criteria aligned with recommended practices, while frequency-based items were categorized such that responses of often and always were considered optimal, and not at all, rarely, and sometimes were considered non-optimal.

Composite scores were primarily reported as continuous measures in the descriptive analysis without categorization. Categorization of scores was applied only for the binary logistic regression analyses, where a 50% cut-off was used to define the dichotomous outcome. This threshold was selected because it represents the point at which a respondent answers more items correctly than incorrectly, providing a clinically interpretable minimum adequacy benchmark, and it has been applied in comparable KAP studies in healthcare settings (15). Sensitivity analyses using continuous outcomes in multivariable linear regression (Supplementary Tables S4–S6) confirmed the robustness of findings to this analytical choice.

### Statistical analysis

Statistical analysis was performed using IBM SPSS Statistics (version 31). GraphPad Prism (version 10) was used for visualization, including the generation of forest plots. Descriptive statistics were used to summarize participant characteristics and study variables. Categorical variables were presented as frequencies and percentages, while continuous variables were summarized using means and standard deviations.

Item-level responses for KAP were described using appropriate descriptive measures. Composite scores for knowledge, attitude, and practice were calculated and reported as continuous variables. Domain-specific scores for attitude and practice were also computed and summarized.

Binary logistic regression analysis was performed to identify factors associated with the study domains. Composite scores were dichotomized using a 50% cut-off to define outcome variables for regression analyses. Independent variables included selected demographic and professional characteristics. Results were reported as odds ratios with 95% confidence intervals. A *p*-value of < 0.05 was considered statistically significant, while *p*-values < 0.1 were considered to indicate borderline statistical significance. Linear regression results are treated as confirmatory; refer to Supplementary Tables S4–S6 alongside each primary logistic regression finding.

Internal consistency reliability was assessed for all KAP scales and their constituent subscales using Cronbach's alpha coefficient. For the knowledge domain, all items were scored as binary (1 = correct, 0 = incorrect); under this condition, Cronbach's alpha is mathematically equivalent to the Kuder–Richardson Formula 20 (KR-20), and no separate computation was therefore required. Reliability was evaluated across the total knowledge scale (67 items), the total attitude scale (28 items), and the total practice scale (14 items), as well as for each domain-level subscale. Results are presented in Supplementary Table S2. Cronbach's alpha values of ≥0.70 were considered acceptable for the purposes of this study, consistent with established benchmarks for health research instruments (16).

## Results

### Participant characteristics

A total of 544 HCPs were included in the analysis (Table 1). Females slightly predominated the sample (53.3%), and most participants were aged 30–40 years (42.6%), followed by those younger than 30 years (29.2%). The majority held a bachelor's degree (73.2%), while 16.4% had postgraduate qualifications and 10.5% had vocational or diploma-level education. General physicians constituted the largest professional group (45.6%), followed by nurses (29.1%) and obstetrics and gynecology specialists (20.2%), with a smaller proportion of family medicine specialists (5.1%). In terms of experience, 38.1% had < 5 years of practice, whereas 21.9% had 20 years or more. Most participants were employed in the public sector (62.5%), and over half were based in the central region of Jordan (54.4%), with the remainder distributed across the northern (30.7%) and southern regions (14.9%). Among respondents reporting income, nearly half were classified as low-income (46.5%), while 38.7% and 14.9% fell into the middle- and high-income categories, respectively.

**Table 1:** Sociodemographic and professional characteristics of the study participants, (*N* = 544).

Category	Sub-category	*N* (%)
Gender		
	Male	254 (46.7)
	Female	290 (53.3)
Age in years		
	< 30	159 (29.2)
	30–40	232 (42.6)
	41–60	109 (20.0)
	≥60	44 (8.1)
Higher educational level		
	Professional (Vocational or Diploma)	57 (10.5)
	Bachelor's	398 (73.2)
	Higher education (Masters or PhD)	89 (16.4)
Professional role		
	General physician	248 (45.6)
	Obstetrics and gynecology specialist	110 (20.2)
	Family medicine specialist	28 (5.1)
	Nurse	158 (29.1)
Years of experience		
	5	207 (38.1)
	5–9	111 (20.4)
	10–19	107 (19.7)
	≥20	119 (21.9)
Work sector		
	Public	340 (62.5)
	Private	96 (17.6)
	Self-employed	108 (19.9)
Location by region		
	North	167 (30.7)
	Middle	296 (54.4)
	South	81 (14.9)
Family monthly income in JOD		
	Not reported	120 (22.1)
	Low ( ≤ 800 JOD)	197 (36.2)
	Middle (801–1,300 JOD)	164 (30.2)
	High (≥1,300 JOD)	63 (11.6)

Percentages may not total 100 due to rounding. Income categories were collapsed based on national economic benchmarks to ensure analytical stability. JOD, Jordanian Dinar. Income data were missing for 120 participants (22.1%); because of missingness, income was not included as a covariate in multivariable models.

### Instrument reliability

Internal consistency was assessed for all KAP scales and subscales prior to analysis (Supplementary Table S2). The total knowledge scale demonstrated excellent reliability (Cronbach's α = 0.937; 67 items). At the subscale level, risk factors (α = 0.917) and signs and symptoms (α = 0.984) showed excellent internal consistency, while mammography recommendations showed questionable but acceptable reliability (α = 0.670). The general knowledge (α = 0.324) and CBE recommendations (α = 0.195) subscales yielded low alpha values, reflecting the heterogeneous factual content of these domains rather than measurement error, as binary knowledge items assessing distinct clinical facts are not expected to be highly intercorrelated; these item indices are therefore interpreted descriptively; corresponding regression analyses are treated as exploratory. The total attitude scale demonstrated good reliability (α = 0.816; 28 items), with subscale values ranging from acceptable to excellent across the five domains (α = 0.683–0.907). The total practice scale showed acceptable reliability (α = 0.773; 14 items); the proper practice frequency subscale demonstrated good internal consistency (α = 0.868), while the proper practice decisions item index yielded lower consistency (α = 0.495), consistent with the heterogeneous clinical scenarios it covers. Composite total scores for all three KAP domains met the threshold for acceptable reliability and were used in subsequent analyses.

### Knowledge of breast cancer early detection and screening

Item-level responses revealed variable knowledge across domains (Table 2). High levels of correct responses were observed for fundamental concepts, including recognizing breast cancer as the most common cancer among women (91.5%), appropriate clinical breast examination (CBE) practices (86.0%), and identification of common signs and symptoms such as breast lumps (81.4%) and nipple discharge (82.0%). Knowledge of several risk factors was also relatively high, particularly family history (82.0%), genetic predisposition (80.9%), and radiation exposure (77.9%). However, notable gaps were identified in more specialized areas, including BIRADS classification (correct responses: 10.5% for BIRADS 3 and 8.6% for BIRADS 5), survival rates in early-stage disease (13.8%), and national screening benchmarks (< 2% correct for early- and late-stage detection rates). Knowledge of screening modalities and recommendations was inconsistent, with lower correct responses for the most effective screening method (41.9%) and the role of ultrasound (38.1%). Furthermore, variation in understanding was observed regarding screening intervals and age-specific recommendations for both CBE and mammography, with correct responses generally below 35% for most age categories.

**Table 2:** Item-level assessment of knowledge regarding breast cancer early detection and screening among healthcare providers, (*N* = 544).

Code	Item	Incorrect, *N* (%)	Correct, *N* (%)
General knowledge			
K1	Breast cancer is the most common cancer in Jordan in both sexes combined	421 (77.4)	123 (22.6)
K2	Breast cancer is the most common cancer in Jordanian women	46 (8.5)	498 (91.5)
K3	Breast cancer develops in women only	68 (12.5)	476 (87.5)
K4	Mammography is painful	216 (39.7)	328 (60.3)
K5	Tamoxifen is the adjuvant endocrine therapy of choice for premenopausal patients with invasive breast cancer	262 (48.2)	282 (51.8)
K6	Treatment of breast cancer affects fertility	249 (45.8)	295 (54.2)
K7	BSE should be done regularly every month	201 (36.9)	343 (63.1)
K8	BSE should start at the age of 25	249 (45.8)	295 (54.2)
K9	BSE technique is inspection and palpation using the palmer side of three middle fingers	61 (11.2)	483 (88.8)
K10	CBE should be done by a trained healthcare provider	76 (14)	468 (86)
K11	In BIRADS 3 biopsy should be considered	487 (89.5)	57 (10.5)
K12	BIRADS 5 is Known biopsy-proven malignancy	497 (91.4)	47 (8.6)
K13	Knowledge of 5-year survival rate in early-stage breast cancer	469 (86.2)	75 (13.8)
K14	Awareness of breast cancer awareness month	272 (50)	272 (50)
K15	Knowledge of percentage of early-stage detection	535 (98.3)	9 (1.7)
K16	Knowledge of percentage of late-stage detection	534 (98.2)	10 (1.8)
K17	Identification of the most effective screening method	316 (58.1)	228 (41.9)
K18	Knowledge of the role of breast ultrasound in screening	337 (61.9)	207 (38.1)
K19	Awareness of national screening and diagnosis guidelines	99 (18.2)	445 (81.8)
Risk factor			
K20	Positive family history	98 (18)	446 (82)
K21	Exposure to radiation	120 (22.1)	424 (77.9)
K22	Not breastfeeding	152 (27.9)	392 (72.1)
K23	Hormone replacement therapy for menopause (HRTs)	159 (29.2)	385 (70.8)
K24	Obesity (BMI >25)	226 (41.5)	318 (58.5)
K25	Alcohol consumption	245 (45)	299 (55)
K26	History of previous benign breast lump	182 (33.5)	362 (66.5)
K27	Recent oral contraceptive use	187 (34.4)	357 (65.6)
K28	Late menopause (>55 years)	197 (36.2)	347 (63.8)
K29	Menarche (>12 years)	252 (46.3)	292 (53.7)
K30	Increasing age	174 (32)	370 (68)
K31	Null parity	171 (31.4)	373 (68.6)
K32	Physical inactivity	259 (47.6)	285 (52.4)
K33	First pregnancy after 32	245 (45)	299 (55)
K34	Smoking	143 (26.3)	401 (73.7)
K35	High fat diet	240 (44.1)	304 (55.9)
K36	Genetics	104 (19.1)	440 (80.9)
K37	Stress	296 (54.4)	248 (45.6)
K38	Large and dense breasts	354 (65.1)	190 (34.9)
K39	Trauma to the breasts	351 (64.5)	193 (35.5)
K40	Giving birth more than four times	278 (51.1)	266 (48.9)
K41	Extended wearing of tight brassieres	357 (65.6)	187 (34.4)
K42	Undergoing mammography	353 (64.9)	191 (35.1)
K43	Being a female	112 (20.6)	432 (79.4)
Signs and symptoms			
K44	Lump in the breast	101 (18.6)	443 (81.4)
K45	Breast redness	145 (26.7)	399 (73.3)
K46	Change in breast color	109 (20)	435 (80)
K47	Nipple discharge or bleeding	98 (18)	446 (82)
K48	Weight loss	128 (23.5)	416 (76.5)
K49	Lump under armpit	99 (18.2)	445 (81.8)
K50	Change in the size the breast	97 (17.8)	447 (82.2)
K51	Change in the shape of the breast	97 (17.8)	447 (82.2)
K52	Pain or soreness in the breast or armpit	154 (28.3)	390 (71.7)
K53	Dimpling of the breast	129 (23.7)	415 (76.3)
K54	Ulceration of the breast	114 (21)	430 (79)
K55	Inversion of/pulling in of the nipple	119 (21.9)	425 (78.1)
K56	Swelling or enlargement of the breast	113 (20.8)	431 (79.2)
K57	Scaling/dry skin on nipple region	150 (27.6)	394 (72.4)
K58	Rash on or around the nipple	147 (27)	397 (73)
K59	Breast develops orange peel	106 (19.5)	438 (80.5)
Clinical breast examination (CBE) recommendations			
K60	Women < 25 years	439 (80.7)	105 (19.3)
K61	Women 25–39 years	364 (66.9)	180 (33.1)
K62	Women 40–49 years	373 (68.6)	171 (31.4)
K63	Women ≥ 50 years	403 (74.1)	141 (25.9)
Mammography recommendations (average-risk women)			
K64	Women < 25 years	242 (44.5)	302 (55.5)
K65	Women 25–39 years	379 (69.7)	165 (30.3)
K66	Women 40–49 years	450 (82.7)	94 (17.3)
K67	Women ≥ 50 years	483 (88.8)	61 (11.2)

Findings from Supplementary Table S3 indicated that participants generally reported moderate to high perceived knowledge across most domains, particularly for breast cancer risk factors and breast self-examination, where over 80% considered themselves moderately or very knowledgeable. In contrast, lower perceived knowledge was reported for more specialized areas, including breast MRI, biopsy, and the BIRADS system, where a considerable proportion of participants rated themselves as slightly or not knowledgeable. Spearman correlation between total perceived knowledge score and total actual knowledge score was weak but statistically significant (*r* = 0.187, 95% CI: 0.102–0.269, *p* < 0.001), suggesting that self-rated knowledge was a poor predictor of objectively measured performance.

### Knowledge score distribution across domains

**Figure 1:**
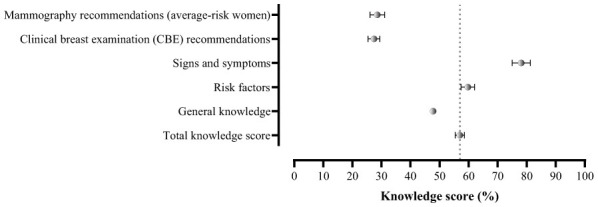
Mean knowledge scores across domains of breast cancer early detection and screening among healthcare providers, (*N* = 544). Dots represent mean knowledge scores expressed as percentages, and horizontal bars represent 95% confidence intervals. The dashed vertical line represents the overall mean total knowledge score of the study population. Knowledge scores were calculated as the proportion of correct responses within each domain.

As shown in Table 3 and Figure 1, the overall mean knowledge score was 57.0% (CI: 55.4–58.6). Domain-specific analysis demonstrated that knowledge was highest for signs and symptoms (78.1%; CI: 75.0–81.2), followed by risk factors (59.8%; CI: 57.4–62.1), while general knowledge scores were comparatively lower (47.8%; CI: 46.9–48.7). It should be noted that the signs and symptoms subscale showed a marked ceiling effect, with a median score of 100% (IQR 78.1–100.0%), indicating that the majority of participants scored at or near the maximum. Because all 67 knowledge items are weighted equally in the composite total score, this ceiling effect is likely to exert an upward influence on the overall mean knowledge score; findings from this subscale should therefore be interpreted as a descriptive summary rather than a discriminating measure of knowledge, and comparisons across groups on this subscale are interpreted with caution. A sensitivity analysis excluding the signs and symptoms subscale reduces the overall mean knowledge score from 57.0% to 50.3%, representing the maximum influence of the ceiling effect on the composite score.

**Table 3:** Summary statistics of knowledge scores across domains of breast cancer early detection and screening among healthcare providers, (*N* = 544).

Codes	Domain	Mean ±SD	95% CI	Median (IQR)
**Total knowledge score**		57.0 ± 19.0	55.4–58.6	64.2 (53.7–68.7)
K1-19	General knowledge	47.8 ± 11.0	46.9–48.7	47.4 (42.1–55.3)
K20-43	Risk factors	59.8 ± 27.4	57.4–62.1	70.8 (54.2–79.2)
K44-59	Signs and symptoms	78.1 ± 37.1	75.0–81.2	100.0 (78.1–100.0)
K60-63	Clinical breast examination (CBE) recommendations	27.4 ± 24.0	25.4–29.5	25.0 (0.0–50.0)
K64-67	Mammography recommendations (average-risk women)	28.6 ± 29.7	26.1–31.1	25.0 (0.0–50.0)

Knowledge scores are expressed as percentages, calculated as the proportion of correct responses out of the total number of items within each domain. Higher scores indicate greater knowledge. SD, standard deviation; CI, confidence interval; IQR, interquartile range.

In contrast, the lowest scores were observed in screening-related domains, with mean scores of 27.4% (CI: 25.4–29.5) for clinical breast examination (CBE) recommendations and 28.6% (CI: 26.1–31.1) for mammography recommendations. These patterns are visually illustrated in Figure 1, where screening-related domains fall substantially below the overall mean knowledge score.

### Attitudes

#### Item-level assessment of attitudes toward breast cancer screening

Item-level responses indicated generally favorable attitudes toward breast cancer prevention and early detection (Table 4). High levels of agreement were observed for the importance of early detection and health promotion, with the majority of participants agreeing or strongly agreeing that breast cancer is curable if detected early (94.7%), that integrating breast cancer promotion into daily practice is important (93.0%), and that distributing educational materials is essential (97.0%). In contrast, perceptions of public awareness were more moderate, with only approximately half of participants agreeing that awareness of breast cancer screening is well established in Jordan. Strong agreement was noted for several patient-level barriers, particularly preferences for female providers (92.6%) and the association with fear of diagnosis, privacy and comfort concerns, and family support on screening uptake. Similarly, structural barriers such as transportation difficulties and the need for increased staffing were widely acknowledged, although fewer participants agreed that screening procedures themselves are difficult. Participants generally reported high levels of confidence in their ability to explain breast cancer and screening modalities, with over 75% expressing agreement or strong agreement across provider readiness items.

**Table 4:** Attitude subscales toward breast cancer screening, (*N* = 544).

		Level of agreement, *N* (%)				
Code	Statement	Strongly disagree	Disagree	Neutral	Agree	Strongly agree
Professional orientation toward breast cancer prevention						
A1	Breast cancer is a high-burden disease	13 (2.4)	67 (12.3)	58 (10.7)	204 (37.5)	202 (37.1)
A2	Breast cancer is curable if detected early	0 (0)	2 (0.4)	27 (5)	188 (34.6)	327 (60.1)
A3	Breast cancer is a major health problem in Jordan	2 (0.4)	33 (6.1)	63 (11.6)	239 (43.9)	207 (38.1)
A4	Integrating breast cancer health promotion in daily practice is important	1 (0.2)	8 (1.5)	29 (5.3)	222 (40.8)	284 (52.2)
A5	Distributing breast cancer education materials is important	1 (0.2)	2 (0.4)	13 (2.4)	195 (35.8)	333 (61.2)
A6	Providing breast cancer counseling to my patients can improve my professional state and increase my professional satisfaction	9 (1.7)	17 (3.1)	65 (11.9)	276 (50.7)	177 (32.5)
Perceived public awareness						
A7	I believe that people in Jordan are well aware of breast cancer screening methods	30 (5.5)	147 (27)	103 (18.9)	177 (32.5)	87 (16)
A8	Awareness of breast cancer is well spread in Jordan	24 (4.4)	121 (22.2)	104 (19.1)	211 (38.8)	84 (15.4)
Perceived patient-level barriers						
A9	Women prefer female healthcare providers to perform screening services	2 (0.4)	7 (1.3)	31 (5.7)	197 (36.2)	307 (56.4)
A10	The fear of the outcome and the treatment stops some women from seeking screening services	2 (0.4)	19 (3.5)	42 (7.7)	274 (50.4)	207 (38.1)
A11	Some women do not feel confident to talk about their symptoms	10 (1.8)	33 (6.1)	47 (8.6)	279 (51.3)	175 (32.2)
A12	Family support is highly important to women seeking screening services	1 (0.2)	3 (0.6)	11 (2)	211 (38.8)	318 (58.5)
A13	Women who do not have symptoms for breast cancer rarely request screening services	5 (0.9)	50 (9.2)	57 (10.5)	271 (49.8)	161 (29.6)
A14	In some cases, women do not seek screening services because they have family obligations	10 (1.8)	40 (7.4)	60 (11)	293 (53.9)	141 (25.9)
A15	Women are embarrassed to undergo screening services	16 (2.9)	36 (6.6)	48 (8.8)	293 (53.9)	151 (27.8)
A16	Women's previous experience in breast examination affects their decision to undergo screening services in the future	14 (2.6)	41 (7.5)	42 (7.7)	308 (56.6)	139 (25.6)
A17	My patients have more pressing health problems than screening	10 (1.8)	92 (16.9)	96 (17.6)	219 (40.3)	127 (23.3)
System and structural barriers						
A18	Screening services are too expensive for my patients	50 (9.2)	133 (24.4)	128 (23.5)	162 (29.8)	71 (13.1)
A19	It is difficult to schedule an appointment for screening services at your facility	50 (9.2)	129 (23.7)	113 (20.8)	178 (32.7)	74 (13.6)
A20	In some cases, women do not undergo screening services due to the difficulty in transportation to reach the health facility	29 (5.3)	79 (14.5)	63 (11.6)	279 (51.3)	94 (17.3)
A21	Increasing staff to increase time for screening services is essential to improve utilization of these services	4 (0.7)	11 (2)	49 (9)	268 (49.3)	212 (39)
A22	The screening procedures are too difficult	71 (13.1)	225 (41.4)	89 (16.4)	118 (21.7)	41 (7.5)
A23	I do not have the capacity to follow-up with patients after screening follow up	51 (9.4)	119 (21.9)	108 (19.9)	182 (33.5)	84 (15.4)
A24	I do not have the necessary equipment or supplies to screen	44 (8.1)	99 (18.2)	114 (21)	183 (33.6)	104 (19.1)
A25	I have not had the necessary training in order to screen	50 (9.2)	79 (14.5)	69 (12.7)	193 (35.5)	153 (28.1)
Provider readiness and self-efficacy						
A26	I feel confident and prepared to explain breast cancer and how it develops to women/men	15 (2.8)	38 (7)	76 (14)	244 (44.9)	171 (31.4)
A27	I feel confident and prepared to explain screening modalities to women/women	12 (2.2)	35 (6.4)	76 (14)	256 (47.1)	165 (30.3)
A28	I feel confident and prepared to discuss the results of the screening modalities to women/men	17 (3.1)	40 (7.4)	91 (16.7)	249 (45.8)	147 (27)

#### Attitude score distribution across domains

**Figure 2:**
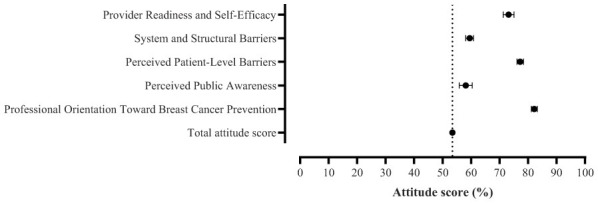
Mean attitude scores across domains of breast cancer early detection and screening among healthcare providers, (*N* = 544). Dots represent mean attitude scores expressed as percentages, and horizontal bars represent 95% confidence intervals. The dashed vertical line represents the overall mean total attitude score of the study population. Attitude scores were calculated based on Likert-scale responses across items within each domain. Higher scores in barrier-related domains indicate greater perceived barriers, whereas higher scores in other domains indicate more favorable attitudes.

As shown in Table 5 and Figure 2, the overall mean attitude score was 53.5% (CI: 52.8–54.1). Domain-specific analysis demonstrated the highest scores for professional orientation toward breast cancer prevention (82.2%; CI: 81.2–83.2), followed by perceived patient-level barriers (77.3%; CI: 76.1–78.4) and provider readiness and self-efficacy (73.2%; CI: 71.3–75.1). Moderate scores were observed for system and structural barriers (59.5%; CI: 58.2–60.9) and perceived public awareness (58.1%; CI: 55.9–60.4). Median values showed a similar distribution across domains. As illustrated in Figure 2, the overall attitude score was positioned near the midpoint, while professional orientation and perceived barriers demonstrated higher domain scores relative to the overall mean.

**Table 5:** Summary statistics of attitude scores across domains of breast cancer early detection and screening among healthcare providers, (*N* = 544).

Codes	Domain	Mean ±SD	95% CI	Median (IQR)
**Total attitude score**		53.5 ± 7.5	52.8–54.1	52.7 (49.1-57.1)
A1-6	Professional orientation toward breast cancer prevention	82.2 ± 12.4	81.2–83.2	83.3 (75.0–91.7)
A7-8	Perceived public awareness	58.1 ± 27.2	55.9–60.4	62.5 (37.5–75.0)
A9-17	Perceived patient-level barriers	77.3 ± 13.3	76.1–78.4	75.0 (69.4–86.1)
A18-25	System and structural barriers	59.5 ± 16.1	58.2–60.9	59.4 (50.0–68.8)
A26-28	Provider readiness and self-efficacy	73.2 ± 22.5	71.3–75.1	75.0 (66.7–91.7)

Attitude scores are expressed as percentages, calculated based on Likert-scale responses across items within each domain. For the total attitude score, items reflecting perceived patient-level and system-level barriers were reverse-coded to ensure that higher scores consistently indicate more favorable attitudes toward breast cancer screening. Domain-specific subscales were calculated using original item coding to preserve construct-specific interpretation. SD, standard deviation; CI, confidence interval; IQR, interquartile range.

### Practice

#### Clinical practice decisions related to breast cancer early detection

As shown in Table 6, the proportion of optimal clinical practice decisions varied across items. High levels of optimal practice were observed for encouraging women to undergo screening (98.9%) and appropriate management of suspected breast cancer cases (91.9%). Areas requiring further support were identified for key screening recommendations, including the correct age to initiate mammography (30.3%), appropriate age limits for screening (24.6%), and recommended screening intervals (20.8%). Moderate levels of optimal practice were reported for encouraging screening practices (34.9%) and follow-up communication with patients (47.1%).

**Table 6:** Correct clinical practice decisions related to breast cancer early detection among healthcare providers (*N* = 544).

Code	Clinical practice decision	Not optimal	Optimal
P1	Correct age to start screening mammography (40 years)	379 (69.7)	165 (30.3)
P2	Appropriate age limit for screening (No age limit)	410 (75.4)	134 (24.6)
P3	Recommended screening interval for an eligible woman	431 (79.2)	113 (20.8)
P4	Encourage screening practices for an eligible woman	354 (65.1)	190 (34.9)
P5	Follow-up communication with patients (telephone, home visits, other ways)	288 (52.9)	256 (47.1)
P6	Appropriate management of suspected breast cancer case	44 (8.1)	500 (91.9)
P7	Encouraging women to undergo breast cancer screening	6 (1.1)	538 (98.9)

Optimal practice indicates responses consistent with breast cancer early detection recommendations as per the guidelines mentioned in the methodology.

#### Frequency of breast cancer early detection practices

The frequency of reported practices is presented in Table 7. A substantial proportion of participants reported frequently or always responding to patient inquiries regarding breast cancer (61.4%) and providing counseling on screening (59.7%). Similarly, 60.5% reported often or always recommending breast self-examination. However, lower frequencies were observed for providing educational materials, with less than one-third reporting doing so often or always (30.0%). Referral to screening programs and teaching breast self-examination showed moderate engagement levels, while performing breast examinations during routine physical assessments was less consistently practiced, with only 35.3% reporting doing so often or always.

**Table 7:** Frequency of breast cancer early detection practices among healthcare providers (*N* = 544).

Code	Practice Situation	Not at all	Rarely	Sometimes	Often	Always
P8	Respond to patient inquiries on breast cancer symptoms and screening	38 (7)	74 (13.6)	98 (18)	95 (17.5)	239 (43.9)
P9	Provide counseling on breast cancer screening	40 (7.4)	65 (11.9)	114 (21)	110 (20.2)	215 (39.5)
P10	Provide breast cancer educational materials	157 (28.9)	102 (18.8)	122 (22.4)	86 (15.8)	77 (14.2)
P11	Refer patients to screening programs	78 (14.3)	79 (14.5)	110 (20.2)	118 (21.7)	159 (29.2)
P12	Recommend breast self-examination	49 (9)	61 (11.2)	105 (19.3)	95 (17.5)	234 (43)
P13	Teach breast self-examination	117 (21.5)	99 (18.2)	111 (20.4)	79 (14.5)	138 (25.4)
P14	Perform breast examination during routine physical exam	144 (26.5)	110 (20.2)	98 (18)	87 (16)	105 (19.3)

#### Practice score distribution across domains

**Figure 3:**
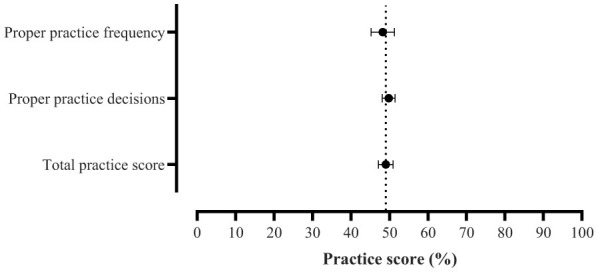
Mean practice scores across domains of breast cancer early detection and screening among healthcare providers, (*N* = 544). Dots represent mean practice scores expressed as percentages, and horizontal bars represent 95% confidence intervals. The dashed vertical line represents the overall mean total practice score of the study population. Practice scores were calculated as the proportion of optimal practice responses within each domain.

As shown in Table 8 and Figure 3, the overall mean practice score was 49.0% (CI: 47.1–50.9). Domain-specific analysis demonstrated comparable performance between proper clinical practice decisions (49.8%; CI: 48.1–51.5) and practice frequency (48.2%; CI: 45.2–51.3). As illustrated in Figure 3, both domains and the overall practice score clustered closely around the midpoint, reflecting moderate levels of adherence to recommended breast cancer early detection practices.

**Table 8:** Summary statistics of practice scores across domains of breast cancer early detection and screening among healthcare providers, (*N* = 544).

Codes	Domain	Mean ±SD	95% CI	Median (IQR)
Total practice score		49 ± 22.4	47.1–50.9	50.0 (28.6–64.3)
P1-7	Proper practice decisions	49.8 ± 20	48.1–51.5	42.9 (28.6–71.4)
P8-14	Proper practice frequency	48.2 ± 36.3	45.2–51.3	50 (14.3–85.7)

Practice scores are expressed as percentages, calculated as the proportion of optimal responses out of the total number of items within each domain. Higher scores indicate more optimal practice. SD, standard deviation; CI, confidence interval; IQR, interquartile range.

#### Predictors of knowledge toward breast cancer screening

Multivariable logistic regression analyses identified several significant predictors of adequate knowledge across domains (Table 9). Female participants had higher odds of adequate knowledge compared to males in total knowledge (OR 1.78; CI: 1.19–2.68) and general knowledge (OR 1.62; CI: 1.00–2.63). Compared with general physicians, nurses were less likely to demonstrate adequate knowledge, particularly in total knowledge (OR 0.46; CI: 0.27–0.79) and mammography recommendations (OR 0.49; CI: 0.27–0.90). Geographic variation was observed, with participants from the southern region showing lower odds of adequate knowledge across multiple domains, including total knowledge (OR 0.46; CI: 0.25–0.86), general knowledge (OR 0.39; CI: 0.21–0.70), risk factors (OR 0.44; CI: 0.24–0.83), and signs and symptoms (OR 0.38; CI: 0.20–0.72), but higher odds in mammography recommendations (OR 1.81; CI: 1.01–3.22). Increasing years of experience were associated with higher odds of adequate knowledge in specific domains, with participants having 10–19 years of experience more likely to demonstrate adequate knowledge in clinical breast examination (CBE) recommendations (OR 2.29; CI: 1.14–4.61), and those with ≥20 years of experience showing higher odds in signs and symptoms knowledge (OR 3.89; CI: 1.17–12.99). Confirmatory multivariable linear regression findings are consistent and presented in Supplementary Table S4. Also, confirmatory Firth penalized likelihood logistic regression for models with suspected quasi-complete separation findings are consistent and presented in Supplementary Table S7.

**Table 9:** Multivariable binary logistic regression analyses of predictors of adequate knowledge toward breast cancer across domains (*N* = 544).

Predictor/*B* (95% CI) and *p*-values	Total knowledge (cut: ≥50%)^‡^	General knowledge (cut: ≥50%)	Risk factors (cut: ≥50%)^†^	Signs and symptoms (cut: ≥50%)	CBE recommendations (cut: ≥50%)^†^	Mammography recommendations (cut: ≥50%)
Gender						
Female (ref: Male)	1.38 (0.86, 2.23)	**1.78 (1.19, 2.68)**	**1.62 (1.00, 2.63)**	1.34 (0.80, 2.23)	1.06 (0.69, 1.63)	1.26 (0.83, 1.90)
	*p* = 0.184	*p* = 0.005^*^	*p* = 0.048^*^	*p* = 0.262	*p* = 0.799	*p* = 0.279
Age group (ref:<30 years)						
30–40 years	1.55 (0.77, 3.11)	0.98 (0.57, 1.72)	1.64 (0.82, 3.30)	1.42 (0.68, 2.99)	1.12 (0.61, 2.04)	0.83 (0.47, 1.49)
	*p* = 0.216	*p* = 0.956	*p* = 0.164	*p* = 0.355	*p* = 0.724	*p* = 0.539
41–60 years	2.47 (0.72, 8.43)	2.22 (0.80, 6.13)	2.48 (0.71, 8.62)	2.27 (0.63, 8.20)	0.69 (0.23, 2.09)	0.42 (0.15, 1.18)
	*p* = 0.150	*p* = 0.124	*p* = 0.154	*p* = 0.213	*p* = 0.510	*p = 0.098* ^†^
Above 60 years	1.66 (0.33, 8.45)	2.05 (0.53, 7.99)	1.46 (0.28, 7.62)	1.47 (0.26, 8.22)	0.36 (0.08, 1.55)	0.48 (0.12, 1.94)
	*p* = 0.543	*p* = 0.302	*p* = 0.651	*p* = 0.659	*p* = 0.168	*p* = 0.304
Education level (ref: diploma/vocational)						
Bachelor's degree	1.06 (0.48, 2.30)	1.59 (0.77, 3.28)	1.26 (0.56, 2.84)	1.10 (0.45, 2.67)	1.93 (0.81, 4.62)	0.95 (0.46, 1.99)
	*p* = 0.892	*p* = 0.212	*p* = 0.581	*p* = 0.833	*p* = 0.137	*p* = 0.898
Postgraduate (Masters/PhD)	0.79 (0.31, 2.01)	1.76 (0.75, 4.13)	0.80 (0.31, 2.07)	0.66 (0.23, 1.83)	1.37 (0.51, 3.71)	1.02 (0.43, 2.41)
	*p* = 0.619	*p* = 0.196	*p* = 0.643	*p* = 0.420	*p* = 0.535	*p* = 0.970
Profession (ref: general physician)						
Family doctor	1.14 (0.39, 3.34)	0.61 (0.26, 1.45)	0.87 (0.31, 2.39)	1.01 (0.34, 3.03)	1.64 (0.71, 3.80)	2.18 (0.95, 5.02)
	*p* = 0.810	*p* = 0.263	*p* = 0.780	*p* = 0.987	*p* = 0.248	*p = 0.066* ^†^
Gynecologist and obstetrician	0.72 (0.38, 1.37)	0.90 (0.53, 1.52)	0.65 (0.35, 1.24)	0.67 (0.34, 1.30)	1.07 (0.61, 1.86)	1.62 (0.95, 2.78)
	*p* = 0.320	*p* = 0.689	*p* = 0.190	*p* = 0.236	*p* = 0.822	*p = 0.077* ^†^
Nurse	0.58 (0.31, 1.06)	**0.46 (0.27, 0.79)**	0.65 (0.35, 1.23)	0.78 (0.39, 1.52)	**0.49 (0.27, 0.90)**	0.72 (0.41, 1.26)
	*p = 0.078* ^†^	*p* = 0.005^*^	*p* = 0.187	*p* = 0.459	*p* = 0.022^*^	*p* = 0.249
Years of experience (ref:<5 years)						
5–9 years	0.99 (0.48, 2.05)	1.13 (0.63, 2.00)	0.92 (0.44, 1.90)	1.28 (0.57, 2.84)	1.25 (0.68, 2.32)	1.64 (0.91, 2.97)
	*p* = 0.969	*p* = 0.688	*p* = 0.816	*p* = 0.551	*p* = 0.472	*p* = 0.102
10–19 years	0.51 (0.22, 1.15)	1.04 (0.53, 2.08)	0.59 (0.26, 1.35)	0.54 (0.23, 1.28)	1.42 (0.69, 2.95)	**2.29 (1.14, 4.61)**
	*p* = 0.103	*p* = 0.902	*p* = 0.209	*p* = 0.164	*p* = 0.344	*p* = 0.020^*^
20+ years	0.59 (0.15, 2.24)	0.62 (0.20, 1.88)	0.73 (0.19, 2.87)	0.77 (0.19, 3.17)	**3.89 (1.17, 12.99)**	2.23 (0.72, 6.94)
		*p* = 0.435	*p* = 0.398	*p* = 0.654	*p* = 0.720	*p* = 0.027^*^	*p* = 0.165
Work sector (ref: private)						
Public sector	1.04 (0.58, 1.87)	0.79 (0.48, 1.30)	1.27 (0.71, 2.27)	1.15 (0.61, 2.14)	0.87 (0.51, 1.48)	0.96 (0.57, 1.61)
	*p* = 0.898	*p* = 0.350	*p* = 0.415	*p* = 0.671	*p* = 0.610	*p* = 0.882
Self-employed	1.13 (0.56, 2.27)	0.88 (0.48, 1.59)	1.45 (0.72, 2.92)	1.22 (0.57, 2.57)	0.65 (0.33, 1.26)	0.86 (0.46, 1.60)
	*p* = 0.744	*p* = 0.662	*p* = 0.303	*p* = 0.611	*p* = 0.200	*p* = 0.627
Geographic region (ref: north)						
Middle region	1.09 (0.67, 1.80)	0.72 (0.49, 1.07)	1.05 (0.63, 1.75)	1.06 (0.61, 1.82)	1.37 (0.88, 2.12)	1.27 (0.83, 1.94)
	*p* = 0.724	*p* = 0.103	*p* = 0.843	*p* = 0.848	*p* = 0.159	*p* = 0.274
South region	**0.46 (0.25, 0.86)**	**0.39 (0.21, 0.70)**	**0.44 (0.24, 0.83)**	**0.38 (0.20, 0.72)**	1.08 (0.58, 2.02)	**1.81 (1.01, 3.22)**
	*p* = 0.015^*^	*p* = 0.002^*^	*p* = 0.011^*^	*p* = 0.003^*^	*p* = 0.812	*p* = 0.045^*^
Model fit statistics						
**Outcome *N* (% adequate)**	430 (79.0%)	231 (42.5%)	433 (79.6%)	448 (82.4%)	158 (29.0%)	183 (33.6%)
**Omnibus *χ*^2^ (df = 16)**	22.07	37.80^*^	25.32	26.76^*^	25.41	24.80
**Omnibus *p*** **-value**	*0.141^‡^*	< 0.001^*^	*0.064* ^†^	0.044^*^	*0.063* ^†^	*0.073* ^†^
**Nagelkerke R^2^**	0.062	0.090	0.071	0.079	0.065	0.062
**Hosmer–Lemeshow *χ*^2^ (df = 8)**	15.01	2.57	8.23	8.72	14.15	4.24
**Hosmer–Lemeshow *p*** **-value**	*0.059* ^†^	0.958	0.411	0.367	*0.078* ^†^	0.835
**AUC**	0.624	0.654	0.628	0.647	0.633	0.619

OR, odds ratio; 95% CI, 95% confidence interval; CBE, clinical breast examination; ref, reference category. Each column represents a separate multivariable binary logistic regression model predicting adequate knowledge (score ≥ 50%) within the specified domain. All models include gender, age group, education level, profession, years of experience, work sector, and geographic region (enter method). Reference categories: Male; age < 30 years; diploma/vocational education; general physician; < 5 years experience; private sector; North region. ^*^p < 0.05 (bold).^†^*p* < 0.10/borderline (italics).^‡^Omnibus *F*-test not significant (*p* ≥ 0.05); model-level results should be interpreted with caution and individual ORs are provided for completeness. Refer to Supplementary Table S4 for linear regression results. The association between ≥20 years of experience and adequate signs and symptoms knowledge [OR 3.89 (1.17–12.99)] was not replicated after Firth penalized likelihood correction (OR 0.80, *p* =0.745), indicating this estimate reflects sparse data rather than a true association. See Supplementary Table S7 for full Firth sensitivity analysis of flagged cells.

#### Predictors of attitudes toward breast cancer screening

Multivariable logistic regression analyses identified several predictors of positive attitudes across domains (Table 10). Female participants had higher odds of positive perceived public awareness compared to males (OR 1.72; CI: 1.12–2.64). Age was associated with specific attitude domains, with participants aged 30–40 years showing a lower likelihood of higher provider readiness and self-efficacy (OR 0.32; CI: 0.11–0.93), and those aged 41–60 years demonstrating substantially lower odds of perceiving patient-level barriers (OR 0.06; CI: 0.01–0.29). Higher educational attainment was associated with more favorable attitudes, as participants with a bachelor's degree had higher odds of positive overall attitudes (OR 2.35; CI: 1.16–4.77) and provider readiness (OR 2.78; CI: 1.10–7.07), while postgraduate participants showed higher odds of provider readiness and self-efficacy (OR 8.22; CI: 1.91–35.42). Regional differences were also observed, with participants from the southern region demonstrating higher odds of positive perceived public awareness (OR 7.96; CI: 3.34–18.97), along with a trend toward higher provider readiness. Additionally, participants with 10–19 years of experience were less likely to report higher perceived patient-level barriers (OR 0.04; CI: 0.002–0.62). Confirmatory multivariable linear regression findings are consistent and presented in Supplementary Table S5. Also, confirmatory Firth penalized likelihood logistic regression for models with suspected quasi-complete separation findings are consistent and presented in Supplementary Table S7.

**Table 10:** Multivariable binary logistic regression analyses of predictors of positive attitudes toward breast cancer screening across domains (*N* = 544).

Predictor/*B* (95% CI) and *p*-values	Total attitude (cut: ≥50%)^‡^	Professiona orientation (NR)	Perceived public awareness (cut: ≥50%)	Patient-level barriers (NR)	System and structural barriers (cut: ≥50%)	Provider readiness and self-efficacy (cut: ≥50%)^§^
Gender						
Female (ref: Male)	1.29 (0.84, 1.99)	*NR*	**1.72 (1.12, 2.64)**	*NR*	0.79 (0.50, 1.27)	1.24 (0.61, 2.52)
	*p* = 0.248		*p* = 0.014^*^		*p* = 0.328	*p* = 0.547
Age group (ref:<30 years)						
30–40 years	1.18 (0.65, 2.14)	*NR*	0.61 (0.34, 1.09)	*NR*	0.87 (0.45, 1.71)	**0.32 (0.11, 0.93)**
	*p* = 0.578		*p = 0.097* ^†^		*p* = 0.689	*p* = 0.035^*^
41–60 years	1.32 (0.43, 4.06)	*NR*	0.65 (0.21, 1.99)	*NR*	1.20 (0.37, 3.91)	**0.06 (0.01, 0.29)**
	*p* = 0.626		*p* = 0.446		*p* = 0.764	*p* < 0.001^*^
Above 60 years	2.09 (0.46, 9.58)	*NR*	1.22 (0.25, 5.97)	*NR*	1.47 (0.31, 6.98)	0.28 (0.02, 4.35)
	*p* = 0.342		*p* = 0.805		*p* = 0.632	*p* = 0.366
Education level (ref: diploma/vocational)						
Bachelor's degree	**2.35 (1.16, 4.77)**	*NR*	0.94 (0.41, 2.14)	*NR*	0.53 (0.22, 1.29)	**2.78 (1.10, 7.07)**
	*p* = 0.018^*^		*p* = 0.884		*p* = 0.163	*p* = 0.031^*^
Postgraduate (Masters/PhD)	2.00 (0.84, 4.76)	*NR*	0.76 (0.29, 1.99)	*NR*	0.39 (0.14, 1.08)	**8.22 (1.91, 35.42)**
	*p* = 0.116		*p* = 0.570		*p = 0.070* ^†^	*p* = 0.005^*^
Profession (ref: general physician)						
Family doctor	1.27 (0.45, 3.62)	*NR*	0.93 (0.37, 2.36)	*NR*	0.48 (0.20, 1.15)	1.16 (0.14, 9.74)
	*p* = 0.655		*p* = 0.884		*p* = 0.101	*p* = 0.892
Gynecologist and obstetrician	0.65 (0.36, 1.15)	*NR*	1.13 (0.63, 2.03)	*NR*	1.13 (0.61, 2.10)	0.47 (0.19, 1.19)
	*p* = 0.139		*p* = 0.681		*p* = 0.706	*p* = 0.110
Nurse	0.80 (0.45, 1.42)	*NR*	1.35 (0.75, 2.41)	*NR*	1.40 (0.74, 2.66)	0.53 (0.20, 1.37)
	*p* = 0.438		*p* = 0.316		*p* = 0.300	*p* = 0.189
Years of experience (ref:<5 years)						
5–9 years	1.08 (0.58, 1.99)	*NR*	1.53 (0.84, 2.80)	*NR*	1.62 (0.78, 3.37)	1.90 (0.69, 5.26)
	*p* = 0.817		*p* = 0.169		*p* = 0.200	*p* = 0.216
10–19 years	1.31 (0.62, 2.76)	*NR*	2.06 (0.99, 4.26)	*NR*	0.64 (0.29, 1.39)	1.98 (0.65, 6.02)
	*p* = 0.486		*p = 0.052* ^†^		*p* = 0.259	*p* = 0.227
20+ years	0.97 (0.29, 3.27)	*NR*	2.38 (0.69, 8.28)	*NR*	0.41 (0.12, 1.47)	4.87 (0.98, 24.26)
	*p* = 0.964		*p* = 0.172		*p* = 0.171	*p = 0.053* ^†^
Work sector (ref: private)						
Public sector	0.74 (0.43, 1.29)	*NR*	0.83 (0.48, 1.44)	*NR*	1.59 (0.93, 2.73)	0.77 (0.33, 1.80)
	*p* = 0.286		*p* = 0.510		*p = 0.091* ^†^	*p* = 0.545
Self-employed	0.77 (0.40, 1.49)	*NR*	1.20 (0.61, 2.37)	*NR*	**3.13 (1.49, 6.55)**	2.11 (0.61, 7.26)
	*p* = 0.443		*p* = 0.590		*p* = 0.002^*^	*p* = 0.237
Geographic region (ref: north)						
Middle region	0.92 (0.60, 1.41)	*NR*	1.44 (0.95, 2.19)	*NR*	0.95 (0.59, 1.53)	0.91 (0.47, 1.79)
	*p* = 0.687		*p = 0.083* ^†^		*p* = 0.841	*p* = 0.794
South region	1.84 (0.93, 3.65)	*NR*	**7.96 (3.34, 18.97)**	*NR*	0.84 (0.44, 1.61)	**12.74 (1.57, 103.21)**
	*p = 0.081* ^†^		*p* < 0.001^*^		*p* = 0.597	*p* = 0.017^*^
Model fit statistics						
**Outcome *N* (% adequate)**	395 (72.6%)	*541^††^ (99.4%)*	377 (69.3%)	*529^††^ (97.2%)*	419 (77.0%)	491 (90.3%)
**Omnibus *χ*^2^ (df = 16)**	19.18	NR	59.92^*^	NR	28.65^*^	58.51^*^
**Omnibus *p*** **-value**	0.260^‡^	NR	< 0.001^*^	NR	0.026^*^	< 0.001^*^
**Nagelkerke *R*^2^**	0.050	NR	0.147	NR	0.078	0.216
**Hosmer–Lemeshow *χ*^2^ (df = 8)**	5.635	NR	11.224	NR	5.937	16.535
**Hosmer–Lemeshow *p***	0.688	NR	0.189	NR	0.654	*0.035^§^*
**AUC**	0.616	0.981	0.697	0.895	0.644	0.797

OR, odds ratio; 95% CI, 95% confidence interval; NR, not reportable. Each column represents a separate multivariable binary logistic regression model predicting a score ≥ 50% within the specified domain. All models adjusted for gender, age group, education level, profession, years of experience, work sector, and geographic region (enter method). Reference categories: Male; age < 30 years; diploma/vocational; general physician; < 5 years; private sector; North region. ^*^p < 0.05 (bold).^†^*p* < 0.10 borderline (italics).^‡^Model omnibus F-test not significant (*p* = 0.260); individual ORs are provided for completeness but should be interpreted with caution.^††^NR columns: Professional Orientation (99.4% adequate) and Patient-Level Barriers (97.2% adequate) show extreme outcome imbalance resulting in quasi-complete separation and model non-convergence; binary logistic regression is not appropriate for these domains. Refer to Supplementary Table S5 for linear regression results. ^§^Provider Readiness model: Hosmer–Lemeshow *p* = 0.035 indicates borderline calibration; extreme outcome imbalance (90.3% adequate) and wide confidence intervals [e.g., South OR 12.74 (1.57–103.21)] reflect sparse data. Results should be interpreted with caution. Note: Models with wide confidence intervals indicative of quasi-complete separation were re-estimated using Firth penalized likelihood logistic regression. Both associations remained statistically significant with attenuated estimates, confirming a real but inflated effect in the primary model. See Supplementary Table S7.

#### Predictors of breast cancer screening practices

Multivariable logistic regression analyses identified several predictors of optimal breast cancer screening practices (Table 11). Female participants had higher odds of optimal overall practice compared to males (OR 1.93; CI: 1.25–2.97). In contrast, nurses were significantly less likely than general physicians to demonstrate optimal practices, both in total practice (OR 0.17; CI: 0.09–0.30) and practice frequency (OR 0.34; CI: 0.19–0.60). Participants working in self-employed settings had higher odds of optimal practice frequency compared to those in the private sector (OR 1.99; CI: 1.07–3.70). Regional differences were also evident, with participants from the southern region showing higher odds of optimal total practice (OR 3.08; CI: 1.64–5.79) and practice frequency (OR 3.25; CI: 1.77–5.96) compared to those from the northern region. Additionally, participants with ≥20 years of experience demonstrated a trend toward higher odds of optimal practice, although this did not reach statistical significance (OR 2.97; CI: 0.92–9.62). Confirmatory multivariable linear regression findings are consistent and presented in Supplementary Table S6. Also, confirmatory Firth penalized likelihood logistic regression for models with suspected quasi-complete separation findings are consistent and presented in Supplementary Table S7.

**Table 11:** Multivariable binary logistic regression analyses of predictors of optimal breast cancer screening practices across domains (*N* = 544).

Predictor/*B* (95% CI) and *p*-values	Total practice (cut: ≥50%)	Practice decisions (NR)	Practice frequency (cut: ≥50%)
Gender			
Female (ref: Male)	**1.93 (1.25, 2.97)**	*NR*	1.45 (0.96, 2.20)
	*p* = 0.003^*^		*p = 0.080* ^†^
**Age group (ref:**<**30 years)**			
30–40 years	1.52 (0.86, 2.69)	*NR*	1.46 (0.84, 2.55)
	*p* = 0.149		*p* = 0.183
41–60 years	0.95 (0.33, 2.71)	*NR*	0.92 (0.33, 2.57)
	*p* = 0.918		*p* = 0.874
Above 60 years	1.01 (0.23, 4.40)	*NR*	0.92 (0.22, 3.81)
	*p* = 0.991		*p* = 0.904
Education level (ref: diploma/vocational)			
Bachelor's degree	0.56 (0.27, 1.16)	*NR*	0.71 (0.35, 1.43)
	*p* = 0.117		*p* = 0.341
Postgraduate (Masters/PhD)	0.59 (0.24, 1.41)	*NR*	0.67 (0.29, 1.58)
	*p* = 0.231		*p* = 0.362
Profession (ref: general physician)			
Family doctor	1.64 (0.64, 4.26)	*NR*	1.12 (0.47, 2.67)
	*p* = 0.305		*p* = 0.796
Gynecologist and obstetrician	1.16 (0.66, 2.03)	*NR*	1.48 (0.85, 2.56)
	*p* = 0.607		*p* = 0.164
Nurse	0.17 (0.09, 0.30)	*NR*	**0.34 (0.19, 0.60)**
	*p* < 0.001^*^		*p* < 0.001^*^
Years of experience (ref:<5 years)			
5–9 years	1.27 (0.71, 2.28)	*NR*	1.22 (0.69, 2.16)
	*p* = 0.428		*p* = 0.504
10–19 years	1.34 (0.66, 2.72)	*NR*	1.78 (0.89, 3.56)
	*p* = 0.422		*p* = 0.102
20+ years	2.97 (0.92, 9.62)	*NR*	2.99 (0.96, 9.38)
	*p = 0.069* ^†^		*p = 0.060* ^†^
Work sector (ref: private)			
Public sector	0.75 (0.45, 1.26)	*NR*	0.88 (0.53, 1.45)
	*p* = 0.277		*p* = 0.606
Self-employed	1.47 (0.78, 2.77)	*NR*	1.99 (1.07, 3.70)
	*p* = 0.240		*p* = 0.030^*^
Geographic region (ref: north)			
Middle region	1.20 (0.79, 1.81)	*NR*	1.28 (0.85, 1.93)
	*p* = 0.388		*p* = 0.231
South region	3.08 (1.64, 5.79)	*NR*	**3.25 (1.77, 5.96)**
	*p* < 0.001^*^		*p* < 0.001^*^
Model fit statistics			
**Outcome *N* (% adequate)**	293 (53.9%)	*239^‡^ (43.9%)*	272 (50.0%)
**Omnibus *χ*^2^ (df=16)**	92.78^*^	NR	72.19^*^
**Omnibus *p*** **-value**	< 0.001^*^	NR	< 0.001^*^
**Nagelkerke *R*^2^**	0.209	NR	0.166
**Hosmer–Lemeshow *χ*^2^ (df = 8)**	5.061	NR	8.182
**Hosmer–Lemeshow *p***	0.751	NR	0.416
**AUC**	0.723	0.813	0.697

OR, odds ratio; 95% CI, 95% confidence interval; NR, Not reportable. Each column represents a separate multivariable binary logistic regression model predicting optimal practice (score ≥ 50%) within the specified domain. All models adjusted for gender, age group, education level, profession (nurse vs. general physician as reference), years of experience, work sector, and geographic region (enter method). Reference categories: Male; age < 30 years; diploma/vocational; general physician; < 5 years; private sector; North region. ^*^p < 0.05 (bold).^†^*p* < 0.10 borderline (italics).^‡^NR—Practice decisions: model did not converge (maximum iterations reached). Quasi-complete separation was detected: nurses score below 50% in practice decisions with near-perfect consistency, rendering binary logistic regression unreliable for this domain (nurse coefficient *B* = −36.6, SE = 4,284.9). Linear regression (Supplementary Table S6) confirms a large, significant nurse effect (*B* = −7.05, *p* < 0.001) and strong education effect (*B* = 5.72, *p* < 0.001). The borderline association between ≥20 years of experience and optimal practice outcomes was evaluated using Firth penalized likelihood logistic regression and remained borderline significant with minimally attenuated estimates (Total Practice OR 2.97 → 2.89; Practice Frequency OR 2.99 → 2.89), supporting its stability. The Practice Decisions domain remained non-reportable after Firth correction, confirming the validity of the NR designation and the use of linear regression as the primary approach for this domain (Supplementary Table S6). See Supplementary Table S7.

## Discussion

This study provides a comprehensive assessment of KAP of HCPs toward breast cancer early detection in Jordan. To our knowledge, this is among the few studies in the region to simultaneously examine all three domains while linking provider performance to system-level factors, including awareness of national screening indicators and implementation constraints. The findings reveal a mixed but highly informative picture; while providers demonstrate strong awareness of breast cancer as a clinical condition and express clear support for early detection, important gaps remain in screening-related knowledge and the consistent application of recommended practices. Notably, this study highlights a critical but underexplored disconnect between clinical knowledge and guideline-driven screening practices, as well as limited integration of national performance indicators into routine care. What emerges is not a lack of willingness, but rather a gap between what providers know, what they believe, and what they are able to implement in practice.

### Knowledge gaps and implications for early detection

There is a well-established mismatch between perceived and actual knowledge. Davis et al., in a landmark systematic review, found that physician self-assessment often showed little or no correlation with objective measures of competence, with the poorest accuracy observed among those who were least skilled and most confident (17). This pattern has also been demonstrated in training settings, where lower-performing residents were more likely to overestimate their abilities (40). More recent evidence suggests that this issue is also relevant to screening-related decisions, Dang et al. (18) found that although confidence was associated with more accurate guideline-concordant decisions, a substantial proportion of confident clinicians still made incorrect choices. A similar confidence–competence imbalance has also been described in other guideline-sensitive clinical domains, where accumulated experience may increase confidence without ensuring equivalent mastery of formal recommendations (19). Consistent with this, correlation analysis in the present study revealed a weak association between perceived and actual knowledge (*r* = 0.187), confirming that self-assessed confidence is a limited proxy for objective performance. Notably, perceived knowledge correlated more strongly with practice (*r* = 0.341), suggesting that provider behavior appears more closely associated with confidence than by guideline-concordant knowledge. This has direct implications for training design: interventions that improve actual knowledge without addressing calibration, the alignment between confidence and competence, may have limited impact on practice change (17, 18). Training programs that increase knowledge without explicitly recalibrating provider confidence against actual competence are therefore unlikely to produce meaningful changes in screening practice.

A closer look at the knowledge domain reveals a clear imbalance. Participants showed strong understanding of signs, symptoms, and several key risk factors, yet their performance dropped considerably when it came to screening recommendations, age-specific guidelines, and national performance indicators. This distinction is important. Recognizing symptomatic disease reflects clinical familiarity, but effective early detection depends on proactive, guideline-driven decision-making, which appears less consistently established. This pattern of stronger clinical knowledge compared to weaker understanding of screening parameters is consistently reported in the literature. Several studies have shown that while HCPs are generally proficient in recognizing signs and symptoms, their knowledge of screening guidelines, eligibility criteria, and modalities remains limited (6, 7). For example, less than half of healthcare workers in some settings were able to correctly identify cancers included in national screening programs or appropriate target age groups, despite being actively involved in patient care (6). Similar gaps have been observed in primary care settings, where providers could recognize clinical presentations but were less certain about screening intervals and program structures (8). This imbalance likely reflects the nature of clinical exposure, where symptom recognition is continuously reinforced through practice, whereas screening recommendations are guideline-driven, periodically updated, and require deliberate engagement (20).

Particularly striking is the very low awareness of national indicators related to early and late-stage detection. This likely reflects a broader issue, namely, that national cancer control metrics are not sufficiently embedded in routine clinical practice or training. Without this linkage, providers may remain disconnected from the larger performance framework that guides early detection programs. At a broader level, limited awareness of national performance indicators appears to reflect a wider implementation gap. Evidence from structured programs such as the National Breast and Cervical Cancer Early Detection Program NBCCEDP in the US suggests that performance improves only when indicators are actively integrated into monitoring systems and professional practice (9, 10). More broadly, this aligns with the well-described challenge in cancer prevention, where substantial evidence exists but is not consistently translated into practice due to system-level and behavioral constraints (21).

Differences across professional groups further reinforce this point. Nurses, in particular, were less likely to demonstrate adequate knowledge compared with physicians, a pattern that is consistent with earlier evidence from Jordan and other settings. In Jordan, another study reported low mean knowledge levels on breast cancer early detection among nurses, while in Ethiopia, a study similarly documented lower knowledge in nursing staff (11, 22). More specifically, Batiston et al. found that physicians were substantially more likely than nurses to correctly identify mammography as the most appropriate screening method, and Seah and Tan showed that nurses' knowledge was generally stronger for basic breast cancer concepts than for screening-related issues (23, 24). Similar findings were also reported by Lemlem et al., who observed that nurses' knowledge of breast cancer and screening was limited and associated with prior training exposure. Given the central role nurses play in frontline care and patient education, these gaps are not trivial; rather, they are likely associated with how screening messages are communicated, interpreted, and reinforced in routine practice (11, 23, 25).

### Attitudes: strong commitment within a constrained environment

In contrast to the variability observed in knowledge, attitudes were consistently positive. Most participants expressed strong agreement with the importance of early detection and the integration of breast cancer awareness into daily clinical practice. This aligns with findings from other settings, including Saudi Arabia and Ethiopia, where HCPs similarly reported favorable attitudes toward breast cancer prevention (12, 22).

At the same time, providers were clearly aware of the challenges their patients face. Concerns related to fear of diagnosis, embarrassment, cultural sensitivity, and the need for female providers were widely reported, alongside structural issues such as transportation barriers, appointment difficulties, and staffing constraints. These observations closely mirror findings from studies conducted in Jordan, Egypt, Bangladesh, and among migrant populations, where gender sensitivity and sociocultural norms play a significant role in shaping screening behavior (26–29).

What stands out here is the coexistence of high motivation and high perceived barriers. Providers appear ready, and even eager, to support early detection, yet they operate within systems that do not always enable them to do so effectively. A similar observation was reported in other previous studies (30). In this sense, the challenge is less about changing attitudes and more about removing the constraints that limit action.

### Practice gaps and the knowledge–practice disconnect

When moving from attitudes to actual practice, the picture becomes more evident. Overall practice levels were moderate, with noticeable variation across different types of activities. Providers reported frequent engagement in communication-based practices, such as counseling and responding to patient inquiries. However, more structured or time-intensive activities, such as teaching breast self-examination, performing routine clinical breast examinations, or providing educational materials, were less consistently implemented.

More concerning are the gaps observed in core screening decisions, including the correct age to initiate screening, appropriate intervals, and eligibility criteria. These are fundamental components of early detection, and deficiencies in these areas suggest that knowledge is not being effectively translated into clinical decision-making. This disconnect between knowledge, attitudes, and practice is well documented in the literature. For instance, Hinz et al. reported that fewer than half of HCPs correctly identified recommended screening ages, despite the vast majority claiming awareness of established guidelines (31). Similarly, Oudsema et al. found that over half of surveyed physicians and trainees felt uncertain about their knowledge of screening recommendations, with notable inconsistencies in decision-making for age groups where guidelines are less uniform (32). Evidence from other settings further reinforces this pattern; a recent study from Brazil showed low adherence to screening protocols and substantial misinterpretation of BI-RADS classifications among both gynecologists and family physicians (33). Even when guidelines are perceived as influential, adherence remains inconsistent, as demonstrated by Nachtigal et al., while broader analyses continue to highlight persistent gaps in translating screening recommendations into practice (34, 35). This reinforces the observed gap, it suggests that improving outcomes will require more than simply increasing awareness. Instead, there is a need to focus on how knowledge is applied, and on the conditions that support, or hinder, its translation into practice.

Because practice was self-reported in a face-to-face workplace setting, the knowledge–practice gap reported here is likely conservative; objectively measured practice would be expected to fall further short of self-report. Accordingly, providers who reported encouraging screening should not be interpreted as a direct measure of clinical behavior.

### Predictors of KAP and implications for targeted interventions

The regression analyses provide additional insight into how KAP outcomes vary across different groups. Female providers were more likely to demonstrate higher knowledge and better practice, which may reflect greater engagement with women's health issues or increased perceived relevance. The consistently lower performance observed among nurses is particularly important. As frontline providers, nurses are often the first point of contact for patients and play a critical role in health education and follow-up. Strengthening their capacity through targeted training and support could therefore have a disproportionately positive impact on screening uptake.

Regional differences also merit attention. Participants from the southern region showed lower knowledge in several domains, yet in some cases demonstrated higher levels of practice. The southern subsample (*n* = 81) is dominated by Al-Karak (*n* = 37, 45.7% of the southern stratum), with smaller contributions from Aqaba (*n* = 21), Ma'an (*n* = 12), and Tafieleh (*n* = 11). Although this pattern may appear counterintuitive, it is supported by plausible explanations in the literature. Targeted outreach efforts led by the Jordan Breast Cancer Program, particularly in southern areas such as Al-Karak, may have contributed to greater provider engagement in screening-related activities, with significant knowledge gains reported following JBCP educational sessions in this region, although findings were context-specific (36). Such initiatives may have facilitated greater provider involvement in counseling, referral, and outreach activities, even without full mastery of screening guidelines. In addition, structural factors may contribute, as providers in underserved regions often assume expanded roles in screening due to limited specialist availability, resulting in higher practice levels consistent with necessity (3). This pattern also reflects the distinction between declarative and procedural knowledge, where providers may effectively carry out screening-related tasks within JBCP-supported or routine service delivery frameworks without fully internalizing guideline-based knowledge. Individual-level training is not tested as a covariate. Residual confounding by training exposure and patient mix cannot be excluded. Differential social-desirability bias by region is also acknowledged as a plausible contributor.

### System and policy implications

The COM-B framework was applied as a *post-hoc* interpretive synthesis and was not a prospective analytical framework for the study design. Mapping these findings onto the COM-B framework clarifies where the binding constraints lie. Psychological capability is the most affected component, with documented gaps in screening-guideline knowledge (CBE/mammography recommendations, BIRADS, expected national indicators). Physical capability shows variable training exposure across professions, particularly nurses and southern-region providers. Physical opportunity is constrained by staffing, equipment, and time limitations reported in the system-barrier sub-scale, while social opportunity is shaped by patient-level cultural barriers (gender preference, embarrassment, family obligations). Motivation, in contrast, is largely supportive, 82.2% of providers report strong professional orientation and broad endorsement of early detection (reflective motivation), with the main automatic-motivation barrier being patients' fear of diagnosis. The implication is that the binding constraint for improving HCP screening practice in Jordan is psychological capability, not motivation, and interventions should accordingly emphasize structured knowledge-based training rather than persuasion or attitude change.

These findings carry important implications for breast cancer control efforts in Jordan. Despite ongoing national initiatives aimed at expanding access to screening and raising awareness, a significant proportion of breast cancer cases continue to be diagnosed at later stages, where outcomes are less favorable and treatment costs are higher (3). The current results suggest that addressing this issue will require a shift in focus. While awareness campaigns and the expansion of service availability remain essential components of national efforts, they are not sufficient on their own. Greater emphasis must be placed on implementation, ensuring that HCPs are adequately equipped, supported, and enabled to deliver screening effectively within their daily practice. This involves strengthening training on screening guidelines, integrating national indicators into routine clinical workflows, enhancing follow-up and referral systems, and addressing structural barriers such as staffing limitations and access challenges. In addition, promoting gender-sensitive service delivery remains critical in improving acceptability and uptake of screening services, particularly within sociocultural contexts where such considerations significantly are associated with health-seeking behavior.

From a behavioral perspective, these findings are consistent with frameworks such as COM-B, where behavior is shaped by capability, opportunity, and motivation. A similar pattern has been documented in breast cancer screening settings, where providers demonstrated high motivation but were constrained by gaps in capability and opportunity, including insufficient training, limited resources, and structural barriers (37). In the present study, the limitation appears to lie primarily in psychological capability, specifically, knowledge of screening guidelines, rather than motivation, which aligns with evidence showing that providers often value screening but lack the detailed knowledge required for consistent implementation (38). Opportunity-related constraints, such as system-level limitations and workflow challenges, likely further compound this gap. This interpretation is further supported by behavior change theory, where deficits in capability and opportunity are recognized as key barriers to translating motivation into action, with targeted education and system-level enablement identified as essential intervention strategies (39).

### Strengths and limitations

This study benefits from a relatively large and diverse sample of HCPs, allowing for a comprehensive assessment across multiple KAP domains. The use of subscales with acceptable internal consistency and multivariable analyses adds robustness to the findings and supports more clear interpretation. At the same time, this study has several limitations that should be considered when interpreting the findings. Cross-sectional design limits the ability to establish causal relationships between KAP domains. Estimates reflect the achieved sample rather than the underlying provider population and should be interpreted accordingly. Although consent among briefed eligible HCPs was essentially complete, the field team did not log providers who were unavailable or not encountered during enumerator visits. Institutional-level exclusions (university hospitals, the Jordanian Royal Medical Services) are likely to bias estimates toward primary-care and public-sector practice patterns. The observed gender associations should be interpreted with caution given the substantial confounding between gender and professional role in the achieved sample. Face-to-face workplace administration is likely to have produced social-desirability inflation of self-reported practice; no objective validation (chart audit, simulated-patient observation, claims data) was conducted. The measurement of KAP domains through composite scores may not fully capture the complexity and variability of real-world clinical behavior. Variations in screening-related items may occur despite that correct responses for screening-related items were defined in accordance with the national breast cancer screening and diagnosis guidelines, which is adapted with permission from the NCCN 1.2017 Breast Cancer Screening and Diagnosis Guidelines (14). Internal consistency reliability varied across domain-specific subscales, with notably lower values observed for the general knowledge, CBE recommendations, and proper practice decisions subscales; findings from these subscales should therefore be interpreted with caution as descriptive summaries rather than psychometrically validated composite scores. Additionally, practice items may not apply equally across all professional categories in terms of scope, especially HCPs assuming administrative or non-clinical roles, so lower scores may partly reflect role-related applicability rather than deficiency alone. Finally, while the study explored multiple domains of KAP, it did not directly assess system-level factors such as workload, resource availability, or institutional policies, which may play a significant role in shaping provider practices.

Taken together, these findings suggest that improving early detection in Jordan is less a question of awareness and more a challenge of implementation, requiring alignment between provider knowledge, system capacity, and structured support mechanisms.

## Conclusion

Overall, the findings suggest that HCPs in Jordan are well-positioned to support breast cancer early detection, with strong motivation and general awareness of its importance. At the same time, the study identifies practical opportunities to strengthen screening-specific knowledge, guideline application, referral practices, and follow-up within routine care. At a national level, these findings can inform targeted, role-specific capacity building, improved integration of national screening guidance into service delivery, and stronger implementation support across healthcare settings, with priority given to nurses. Training content should target knowledge areas related to mammography age and interval guidelines, BIRADS classification, CBE frequency recommendations, and national early-detection performance benchmarks. Strengthening these areas can contribute to earlier detection, improved patient navigation, and continued progress in reducing the burden of breast cancer in Jordan.

## Data Availability

The datasets generated and/or analyzed during the current study are available from the corresponding author on reasonable request.
