# Supplementary material for: Knowledge, attitudes, and practice in breast cancer early detection: evidence from a nationally stratified study of healthcare providers in Jordan
Source: Front Public Health. 2026 Jul 22;14:1845992. doi: 10.3389/fpubh.2026.1845992 (PMC13437638; doi:10.3389/fpubh.2026.1845992)
Supplement: Supplementary file 1 [file Supplementary_File_1.docx]

**Supplementary Table S1. Population frame and planned sample (N = 544).**

| **Specialty** | **MOH 2024 population** | **Population weight** | **Planned sample** | **Adjusted sample** | **Achieved (manuscript)** |
| --- | --- | --- | --- | --- | --- |
| General Physician | 25,347 | 44.0% | 231 | 180 | 248 |
| Family Medicine Specialist | 564 | 1.0% | 5 | 50 | 28 |
| Community Medicine | 134 | 0.2% | 1 | 50 | 0 (excluded) |
| Obstetrics and gynecology specialist (OB-GYN) | 1,603 | 2.8% | 15 | 50 | 110 |
| Nurses and midwives* | 30,000 | 52.0% | 273 | 195 | 158 |
| Total | 57,648 | 100% | 525 | 525 | 544 |

* Midwives included are only two and were labeled as nurses in the analysis.

**Supplementary Table S2. Reliability assessment of Knowledge, Attitudes, and Practice scales and subscales (N = 544).**

| **Scale / Subscale** | **Item Codes** | **N Items** | **Cronbach's α** | **Interpretation** |
| --- | --- | --- | --- | --- |
| **Total Knowledge Scale** | **K1–K67** | **67** | **0.937** | **Excellent** |
| General knowledge | K1–K19 | 19 | 0.324 | Unacceptable |
| Risk factors | K20–K43 | 24 | 0.917 | Excellent |
| Signs and symptoms | K44–K59 | 16 | 0.984 | Excellent |
| CBE recommendations | K60–K63 | 4 | 0.195 | Unacceptable |
| Mammography recommendations (average-risk women) | K64–K67 | 4 | 0.670 | Questionable |
| **Total Attitude Scale** | **A1–A28** | **28** | **0.816** | **Good** |
| Professional orientation toward breast cancer prevention | A1–A6 | 6 | 0.683 | Questionable |
| Perceived public awareness | A7–A8 | 2 | 0.880 | Good |
| Perceived patient-level barriers | A9–A17 | 9 | 0.791 | Acceptable |
| System and structural barriers | A18–A25 | 8 | 0.685 | Questionable |
| Provider readiness and self-efficacy | A26–A28 | 3 | 0.907 | Excellent |
| **Total Practice Scale** | **P1–P14** | **14** | **0.773** | **Acceptable** |
| Proper practice decisions | P1–P7 | 7 | 0.495 | Unacceptable |
| Proper practice frequency | P8–P14 | 7 | 0.868 | Good |

Cronbach's alpha (α) ≥ 0.90 = Excellent; 0.80–0.89 = Good; 0.70–0.79 = Acceptable; 0.60–0.69 = Questionable; 0.50–0.59 = Poor; < 0.50 = Unacceptable. All knowledge items were scored as binary (1 = correct, 0 = incorrect). All attitude items used a 5-point Likert scale (1 = Strongly disagree to 5 = Strongly agree). Proper practice decision items were scored as binary (1 = optimal, 0 = not-optimal), and Proper practice frequency items used a 5-point Likert scale (1 = Not at all to 5 = Always). Subscales with α<0.50 are treated as heterogeneous item indices and interpreted descriptively; corresponding regressions are exploratory.

**Supplementary Table S3. Item-level assessment of perceived knowledge regarding breast cancer early detection and screening among healthcare providers, and correlation with objective knowledge and practice scores (N = 544).**

|  | Perception of knowledge, N(%) | | | |
| --- | --- | --- | --- | --- |
|  | Not knowledgeable at all | Slightly Knowledgeable | Moderately Knowledgeable | Very Knowledgeable |
| Breast cancer (risk factors, symptoms, and treatment) | 5 (0.9) | 40 (7.4) | 293 (53.9) | 206 (37.9) |
| Breast Self-Examination (technique, recommended age group, frequency) | 6 (1.1) | 45 (8.3) | 236 (43.4) | 257 (47.2) |
| Clinical breast examination (technique, recommended age group, frequency) | 15 (2.8) | 67 (12.3) | 226 (41.5) | 236 (43.4) |
| Mammography (technique, recommended age group, frequency) | 60 (11) | 161 (29.6) | 208 (38.2) | 115 (21.1) |
| Breast ultrasound (technique, indication) | 73 (13.4) | 158 (29) | 199 (36.6) | 114 (21) |
| Breast biopsy (technique, indication) | 96 (17.6) | 195 (35.8) | 175 (32.2) | 78 (14.3) |
| Breast MRI (technique, indication) | 96 (17.6) | 205 (37.7) | 170 (31.3) | 73 (13.4) |
| BIRADs system | 186 (34.2) | 172 (31.6) | 121 (22.2) | 65 (11.9) |
| Breast cancer risk assessment | 26 (4.8) | 54 (9.9) | 255 (46.9) | 209 (38.4) |
| Total perceived knowledge score |  | **Mean ± SD** | **Minimum** | **Maximum** |
|  |  | 45.6 ± 15.3% | 2.8% | 75.0% |
| Correlation analysis |  | **Spearman's r** | **95% CI** | **p-value** |
| Total perceived knowledge vs. total actual knowledge score | | 0.187 | 0.102–0.269 | <0.001 |
| Total perceived knowledge vs. total practice score | | 0.341 | 0.262–0.415 | <0.001 |

Perceived knowledge was assessed using a 4-point scale (1 = not knowledgeable at all; 4 = very knowledgeable). The total perceived knowledge score was computed as the mean of all nine items, rescaled to a percentage (0–100%). Spearman's rho was used to assess the association between total perceived knowledge and both total actual knowledge and total practice scores. The weak correlation between perceived and actual knowledge (r = 0.187) indicates limited alignment between self-assessed and objectively measured competence. The comparatively stronger correlation with practice scores (r = 0.341) suggests that provider confidence may be a more proximal driver of screening-related behavior than actual knowledge. It should be noted that the correlations presented here compare aggregate total scores. While a more granular approach matching individual perceived knowledge items to their corresponding actual knowledge domains, such as perceived CBE knowledge against the CBE recommendations subscale, would provide finer-grained insight into domain-specific miscalibration, this level of analysis was constrained by the partial structural overlap between the two item sets, as not all perceived knowledge items have a directly equivalent scored domain in the actual knowledge scale. Domain-level alignment between perceived and objective knowledge represents a meaningful direction for future research.

**Supplementary Table S4. Sensitivity Analysis: Multivariable Linear Regression of Knowledge Domain Scores (Continuous) on Demographic and Professional Predictors (N = 544)**

| **Predictor/**  B (95% CI) and p-values | **Total Knowledge**  **Score (%)** | **General Knowledge**  **Score (%)** | **Risk Factors**  **Score (%)** | **Signs & Symptoms**  **Score (%) ‡** | **CBE Recommendations**  **Score (%) ‡** | **Mammography**  **Score (%) §** |
| --- | --- | --- | --- | --- | --- | --- |
| **Gender (Female vs. Male)** | 3.43 (−0.08, 6.93)  *p = 0.055†* | **4.03 (2.05, 6.01)**  **p < 0.001*** | 3.95 (−1.12, 9.01)  p = 0.127 | 3.14 (−3.76, 10.03)  p = 0.372 | −0.09 (−4.55, 4.37)  p = 0.969 | 2.09 (−3.42, 7.61)  p = 0.456 |
| **Age group** | 1.40 (−2.04, 4.84)  p = 0.424 | **1.97 (0.02, 3.92)**  **p = 0.048*** | 1.19 (−3.78, 6.17)  p = 0.638 | 2.96 (−3.81, 9.74)  p = 0.390 | −1.93 (−6.32, 2.45)  p = 0.387 | −2.96 (−8.37, 2.46)  p = 0.284 |
| **Education level** | −0.82 (−4.08, 2.44)  p = 0.622 | **2.99 (1.14, 4.83)**  **p = 0.002*** | −2.29 (−7.01, 2.43)  p = 0.341 | −5.09 (−11.51, 1.33)  p = 0.120 | 1.42 (−2.73, 5.58)  p = 0.501 | 4.76 (−0.38, 9.89)  *p = 0.069†* |
| **Profession: Nurse**  **(vs. General Physician)** | **−1.87 (−3.30, −0.43)**  **p = 0.011*** | **−1.28 (−2.10, −0.47)**  **p = 0.002*** | **−2.27 (−4.35, −0.19)**  **p = 0.033*** | −2.10 (−4.93, 0.73)  p = 0.145 | **−1.89 (−3.72, −0.06)**  **p = 0.043*** | −1.27 (−3.53, 0.99)  p = 0.271 |
| **Years of experience** | −0.69 (−3.37, 2.00)  p = 0.616 | −0.56 (−2.08, 0.96)  p = 0.466 | −1.11 (−4.99, 2.78)  p = 0.575 | −2.11 (−7.40, 3.18)  p = 0.434 | 3.04 (−0.38, 6.47)  *p = 0.081†* | 3.21 (−1.02, 7.44)  p = 0.136 |
| **Work sector** | 0.60 (−1.08, 2.29)  p = 0.483 | −0.02 (−0.98, 0.93)  p = 0.962 | 1.47 (−0.96, 3.91)  p = 0.236 | 0.65 (−2.67, 3.97)  p = 0.700 | −1.72 (−3.87, 0.42)  p = 0.115 | 0.49 (−2.16, 3.14)  p = 0.718 |
| **Geographic region** | **−3.29 (−5.72, −0.85)**  **p = 0.008*** | **−1.78 (−3.16, −0.41)**  **p = 0.011*** | **−5.00 (−8.52, −1.48)**  **p = 0.005*** | **−5.67 (−10.46, −0.88)**  **p = 0.020*** | 1.38 (−1.73, 4.48)  p = 0.384 | **4.69 (0.86, 8.52)**  **p = 0.017*** |
| **Model Fit Statistics** |  |  |  |  |  |  |
| **R²** | 0.032 | 0.075 | 0.033 | 0.021 | 0.019 | 0.024 |
| **Adjusted R²** | 0.019 | 0.063 | 0.020 | 0.009 | 0.006 | 0.012 |
| **F (df = 7, 536)** | 2.53 | 6.19 | 2.61 | 1.68 | 1.47 | 1.91 |
| **ANOVA p-value** | **0.015*** | **<0.001*** | **0.012*** | 0.112 | 0.176 | *0.066†* |

Abbreviations: B = unstandardized regression coefficient; 95% CI = 95% confidence interval; CBE = clinical breast examination; df = degrees of freedom. All models adjusted for gender, age group, education level, profession (nurse vs. general physician), years of experience, work sector, and geographic region, using the enter method. Total scores are expressed as a percentage (0–100%). B coefficients therefore reflect the expected change in the respective score unit per one-unit increase in each predictor. * Statistically significant at p < 0.05 (bold). † Borderline significant at p < 0.10 (italics). ‡ Model omnibus F-test not significant (p ≥ 0.05); individual coefficients shown for completeness and should be interpreted with caution. § Model omnibus F-test borderline significant (p = 0.066). Results are presented as a sensitivity analysis to complement the primary binary logistic regression analyses (Table 9 in the main manuscript), addressing the concern that the 50% cut-off used for dichotomization may introduce analytical artefacts. Consistency of significant predictors across both analytical approaches supports the robustness of the primary findings. Collinearity diagnostics (identical across all models): Gender VIF = 1.22, Age VIF = 3.85, Education VIF = 1.13, Profession VIF = 1.42, Years of experience VIF = 4.01, Work sector VIF = 1.05, Geographic region VIF = 1.02. All VIF values < 5, indicating no problematic multicollinearity.

**Supplementary Table S5. Sensitivity Analysis: Multivariable Linear Regression of Attitude Domain Scores (Continuous) on Demographic and Professional Predictors (N = 544).**

| **Predictor/**  B (95% CI) and p-values | **Total Attitude**  **Score (%)** | **Professional**  **Orientation (%)** | **Perceived Public**  **Awareness (%)** | **Patient-Level**  **Barriers (%)‡** | **System &**  **Structural**  **Barriers (%)‡** | **Provider Readiness**  **& Self-Efficacy (%)** |
| --- | --- | --- | --- | --- | --- | --- |
| **Gender (Female vs. Male)** | 1.25 (−0.11, 2.62)  *p = 0.071†* | **3.54 (1.26, 5.81)**  **p = 0.002*** | **6.86 (2.11, 11.62)**  **p = 0.005*** | 0.85 (−1.61, 3.31)  p = 0.498 | 0.19 (−2.79, 3.17)  p = 0.901 | 0.76 (−3.35, 4.87)  p = 0.717 |
| **Age group** | 0.58 (−0.76, 1.92)  p = 0.396 | 1.93 (−0.30, 4.17)  *p = 0.089†* | 0.67 (−4.00, 5.34)  p = 0.778 | 1.29 (−1.12, 3.71)  p = 0.294 | −1.09 (−4.02, 1.84)  p = 0.466 | −0.75 (−4.79, 3.29)  p = 0.714 |
| **Education level** | **1.37 (0.10, 2.64)**  **p = 0.034*** | −1.18 (−3.30, 0.94)  p = 0.274 | −1.85 (−6.28, 2.58)  p = 0.413 | −2.25 (−4.55, 0.04)  *p = 0.054†* | −2.35 (−5.13, 0.43)  *p = 0.097†* | **5.57 (1.75, 9.40)**  **p = 0.004*** |
| **Profession: Nurse**  **(vs. General Physician)** | −0.43 (−0.99, 0.13)  p = 0.133 | −0.87 (−1.80, 0.06)  *p = 0.067†* | 0.78 (−1.17, 2.73)  p = 0.431 | −0.13 (−1.13, 0.89)  p = 0.808 | 0.20 (−1.02, 1.43)  p = 0.747 | **−2.33 (−4.01, −0.64)**  **p = 0.007*** |
| **Years of experience** | 0.57 (−0.48, 1.61)  p = 0.285 | 0.71 (−1.04, 2.45)  p = 0.427 | **4.77 (1.13, 8.42)**  **p = 0.010*** | −0.66 (−2.55, 1.23)  p = 0.493 | 0.55 (−1.74, 2.84)  p = 0.636 | 1.26 (−1.89, 4.41)  p = 0.433 |
| **Work sector** | −0.46 (−1.11, 0.20)  p = 0.172 | 0.11 (−0.98, 1.21)  p = 0.838 | 1.10 (−1.19, 3.39)  p = 0.345 | 0.13 (−1.05, 1.32)  p = 0.827 | **2.35 (0.91, 3.78)**  **p = 0.001*** | **2.33 (0.36, 4.31)**  **p = 0.021*** |
| **Geographic region** | **1.23 (0.28, 2.18)**  **p = 0.011*** | −1.38 (−2.95, 0.21)  *p = 0.088†* | **9.41 (6.11, 12.72)**  **p < 0.001*** | **−2.94 (−4.65, −1.23)**  **p < 0.001*** | −0.53 (−2.60, 1.55)  p = 0.618 | −0.63 (−3.49, 2.23)  p = 0.665 |
| **Model Fit Statistics** |  |  |  |  |  |  |
| **R²** | 0.051 | 0.050 | 0.132 | 0.031 | 0.026 | 0.053 |
| **Adjusted R²** | 0.039 | 0.037 | 0.121 | 0.018 | 0.013 | 0.040 |
| **F (df = 7, 536)** | **4.15*** | **4.00*** | **11.68*** | **2.45*** | **2.04*** | **4.25*** |
| **ANOVA p-value** | **<0.001*** | **<0.001*** | **<0.001*** | **0.017*** | **0.049*** | **<0.001*** |

B = unstandardized regression coefficient; 95% CI = 95% confidence interval. All models use the enter method with the same 7 predictors. Age, years of experience, and work sector are entered as continuous ordinal-coded variables; B reflects change per unit increase. All attitude scores are expressed as percentages. The Total Attitude score uses reverse-coded barrier items (higher = more favorable). Domain-specific subscales use original item coding: for patient-level and system/structural barrier domains, higher scores reflect greater perceived barriers. ‡ Barrier domains (Patient-Level Barriers, System & Structural Barriers): B coefficients reflect associations with raw barrier scores (higher = more perceived barriers). Direction is not reverse-coded in these subdomain regressions. * p < 0.05 (bold). † p < 0.10 borderline (italics). All ANOVA F-tests significant (p < 0.05); individual estimates provided as sensitivity analysis to primary binary logistic regression models (Table 10), addressing the concern about the 50% dichotomization threshold. Collinearity diagnostics (identical across all models): Gender VIF = 1.22, Age VIF = 3.85, Education VIF = 1.13, Profession VIF = 1.42, Years of experience VIF = 4.01, Work sector VIF = 1.05, Geographic region VIF = 1.02. All VIF values < 5, indicating no problematic multicollinearity.

**Supplementary Table S6. Sensitivity Analysis: Multivariable Linear Regression of Practice Domain Scores (Continuous) on Demographic and Professional Predictors (N = 544).**

| **Predictor/**  B (95% CI) and p-values | **Total Practice**  **Score (%)** | **Practice Decisions**  **Score (points)‡** | **Practice Frequency**  **Score (points)** |
| --- | --- | --- | --- |
| **Gender (Female vs. Male)** | **5.54 (1.69, 9.40)**  **p = 0.005*** | **3.37 (0.11, 6.63)**  **p = 0.043*** | **7.72 (1.29, 14.15)**  **p = 0.019*** |
| **Age group** | **4.36 (0.57, 8.16)**  **p = 0.024*** | 2.40 (−0.80, 5.61)  p = 0.141 | **6.32 (0.01, 12.64)**  **p = 0.050*** |
| **Education level** | **4.12 (0.52, 7.71)**  **p = 0.025*** | **5.72 (2.68, 8.76)**  **p < 0.001*** | 2.51 (−3.48, 8.50)  p = 0.411 |
| **Profession: Nurse**  **(vs. General Physician)** | **−5.71 (−7.29, −4.13)**  **p < 0.001*** | **−7.05 (−8.39, −5.71)**  **p < 0.001*** | **−4.37 (−7.01, −1.74)**  **p = 0.001*** |
| **Years of experience** | 0.98 (−1.98, 3.94)  p = 0.515 | −1.17 (−3.67, 1.34)  p = 0.361 | 3.13 (−1.80, 8.06)  p = 0.213 |
| **Work sector** | 1.46 (−0.40, 3.32)  p = 0.123 | −0.94 (−2.51, 0.63)  p = 0.242 | **3.85 (0.76, 6.94)**  **p = 0.015*** |
| **Geographic region** | **3.66 (0.98, 6.35)**  **p = 0.008*** | −0.27 (−2.54, 2.00)  p = 0.814 | **7.60 (3.13, 12.06)**  **p < 0.001*** |
| **Model Fit Statistics** |  |  |  |
| **R²** | 0.158 | 0.244 | 0.109 |
| **Adjusted R²** | 0.147 | 0.234 | 0.097 |
| **F (df = 7, 536)** | **14.39*** | **24.72*** | **9.35*** |
| **ANOVA p-value** | **<0.001*** | **<0.001*** | **<0.001*** |

B = unstandardized regression coefficient; 95% CI = 95% confidence interval. All models use the enter method with identical predictor set. Age, experience, education, and work sector entered as ordinal-coded variables; B reflects change per unit increase. Total practice score is expressed as a percentage (0–100%). Practice decision and frequency domain scores are expressed as raw sums (points). B coefficients reflect the expected change per unit increase in each predictor. ‡ Practice Decisions: binary logistic regression was not reportable due to quasi-complete separation (nurses virtually universally score below 50%, creating a near-perfect predictor). The linear regression above is the primary valid approach for this domain, confirming large and highly significant associations for nurse profession (B = −7.05, p < 0.001) and education (B = 5.72, p < 0.001). * p < 0.05 (bold). † p < 0.10 borderline (italics). All ANOVA F-tests significant (p < 0.05). These results provide sensitivity analysis for Table 11, confirming robustness of findings to the choice of the 50% dichotomization threshold. Collinearity diagnostics (identical across all models): Gender VIF = 1.22, Age VIF = 3.85, Education VIF = 1.13, Profession VIF = 1.42, Years of experience VIF = 4.01, Work sector VIF = 1.05, Geographic region VIF = 1.02. All VIF values < 5, indicating no problematic multicollinearity.

**Supplementary Table S7. Sensitivity Analysis: Firth Penalized Likelihood Logistic Regression for Models with Suspected Quasi-Complete Separation (N = 544).**

| **Flagged predictor** | **Outcome domain** | **Original OR [95% CI], p** | **Firth OR [95% CI], p** |
| --- | --- | --- | --- |
| **Knowledge domain** | | | |
| Experience ≥20 years vs <5 years | Signs & Symptoms knowledge | **3.89 [1.17–12.99]**  **p = .027*** | 0.80 [0.20–3.09]  p = .745 |
| **Attitude domain — Perceived Public Awareness** | | | |
| South vs North region | Perceived Public Awareness | **7.96 [3.34–18.97]**  **p < .001*** | **7.15 [3.26–17.82]**  **p < .001*** |
| **Attitude domain — Patient-Level Barriers (primary model: NR — 97.2% adequate)** | | | |
| Experience 10–19 years vs <5 years | Patient-Level Barriers | NR† | **0.064 [0.004–0.595]**  **p = .013*** |
| South vs North region | Patient-Level Barriers | NR† | **0.031 [0.0002–0.261]**  **p < .001*** |
| **Attitude domain — Provider Readiness & Self-Efficacy** | | | |
| Age 30–40 vs <30 years | Provider Readiness & Self-Efficacy | **0.32 [0.11–0.93]**  **p = .035*** | **0.34 [0.12–0.93]**  **p = .035*** |
| Age 41–60 vs <30 years | Provider Readiness & Self-Efficacy | **0.06 [0.01–0.29]**  **p < .001*** | **0.07 [0.015–0.318]**  **p < .001*** |
| Bachelor's degree vs Diploma | Provider Readiness & Self-Efficacy | **2.78 [1.10–7.07]**  **p = .031*** | **2.65 [1.07–6.57]**  **p = .035*** |
| Postgraduate vs Diploma | Provider Readiness & Self-Efficacy | **8.22 [1.91–35.42]**  **p = .005*** | **6.84 [1.82–30.07]**  **p = .004*** |
| Experience ≥20 years vs <5 yrs | Provider Readiness & Self-Efficacy | 4.87 [0.98–24.26]  p = .053† | 4.50 [0.96–21.37]  p = .056† |
| South vs North region | Provider Readiness & Self-Efficacy | **12.74 [1.57–103.21]**  **p = .017*** | **8.25 [1.90–77.87]**  **p = .003*** |
| **Practice domain — Practice Decisions (primary model: NR — near-complete separation)** | | | |
| Nurse vs General Physician | Practice Decisions | NR (B = −36.6, SE = 4284.9) | **<0.01 [<0.001–0.013]**  **p < .001*** |
| **Practice domain — Practice Frequency** | | | |
| Experience ≥20 years vs <5 years | Practice Frequency | 2.99 [0.96–9.38]  p = .060† | 2.89 [0.95–8.89]  p = .063† |

OR = odds ratio; CI = 95% confidence interval; NR = not reportable; Firth = Firth penalized maximum likelihood estimation. * p < 0.05 (bold, dark red). † borderline p < 0.10 (italics). Only predictors flagged as suspected quasi-complete separation based on CI width ratio ≥8× or non-convergence in the primary analysis are shown. † Patient-Level Barriers primary model was designated NR (not reportable) due to extreme outcome imbalance (97.2% adequate score ≥50%), resulting in near-complete separation and model non-convergence. Firth results are presented here for completeness only and confirm that extreme imbalance persists. ‡ Practice Decisions primary model was designated NR due to quasi-complete separation (nurses score below 50% with near-perfect consistency; B = −36.6, SE = 4284.9). Firth correction does not resolve this separation (OR <0.01), confirming that linear regression (Supplementary Table S6) remains the only valid approach for this domain. Convergence note: All Firth models returned 'FAILED' convergence under the default profile log-likelihood criterion (1×10⁻⁶); last log-likelihood changes ranged from 5×10⁻⁶ to 9×10⁻⁶. Estimates were identical across runs with 100 to 1000 iterations, confirming substantive stability. The non-convergence flag reflects a software tolerance threshold and does not indicate algorithmic instability. Key finding: The signs and symptoms × ≥20 years experience association (originally OR 3.89 [1.17–12.99]*) was not replicated after Firth correction (OR 0.80, p=.745), confirming it was a separation artifact in the primary model.

**Supplementary Table S8. Sociodemographic and professional characteristics of healthcare providers in the southern region in Jordan (N = 81).**

| **Characteristic** | **South region, N(%)** |
| --- | --- |
| **Gender** | |
| Male | 44 (54.3%) |
| Female | 37 (45.7%) |
| **Professional Role** | |
| General Physician | 36 (44.4%) |
| Nurse | 23 (28.4%) |
| Gynecologist & Obstetrician | 15 (18.5%) |
| Family Medicine Specialist | 7 (8.6%) |
| **Work Sector** | |
| Public | 51 (63.0%) |
| Private | 16 (19.8%) |
| Self-Employed | 14 (17.3%) |

Values are reported as N (%). The southern region comprises four governorates: Al-Karak (n=37, 45.7%), Aqaba (n=21, 25.9%), Ma'an (n=12, 14.8%), and Tafileh (n=11, 13.6%).
